# A Defective TLR4 Signaling for IFN-β Expression Is Responsible for the Innately Lower Ability of BALB/c Macrophages to Produce NO in Response to LPS as Compared to C57BL/6

**DOI:** 10.1371/journal.pone.0098913

**Published:** 2014-06-09

**Authors:** Luciana S. Oliveira, Nina M. G. P. de Queiroz, Laura V. S. Veloso, Thaís G. Moreira, Fernanda S. Oliveira, Matheus B. H. Carneiro, Ana M. Faria, Leda Q. Vieira, Sérgio C. Oliveira, Maria F. Horta

**Affiliations:** 1 Departamento de Bioquímica e Imunologia, Instituto de Ciências Biológicas Universidade Federal de Minas Gerais, 31270-901 Belo Horizonte, MG, Brazil; 2 Departamento de Alimentos, Faculdade de Farmácia, Universidade Federal de Minas Gerais, 31270-901 Belo Horizonte, MG, Brazil; National Jewish Health and University of Colorado School of Medicine, United States of America

## Abstract

C57BL/6 mice macrophages innately produce higher levels of NO than BALB/c cells when stimulated with LPS. Here, we investigated the molecular events that account for this intrinsic differential production of NO. We found that the lower production of NO in BALB/c is not due to a subtraction of L-arginine by arginase, and correlates with a lower iNOS accumulation, which is independent of its degradation rate. Instead, the lower accumulation of iNOS is due to the lower levels of iNOS mRNA, previously shown to be also independent of its stability, suggesting that *iNOS* transcription is less efficient in BALB/c than in C57BL/6 macrophages. Activation of NFκB is more efficient in BALB/c, thus not correlating with iNOS expression. Conversely, activation of STAT-1 does correlate with iNOS expression, being more prominent in C57BL/6 than in BALB/c macrophages. IFN-β and IL-10 are more highly expressed in C57BL/6 than in BALB/c macrophages, and the opposite is true for TNF-α. Whereas IL-10 and TNF-α do not seem to participate in their differential production of NO, IFN-β has a determinant role since 1) anti-IFN-β neutralizing antibodies abolish STAT-1 activation reducing NO production in C57BL/6 macrophages to levels as low as in BALB/c cells and 2) exogenous rIFN-β confers to LPS-stimulated BALB/c macrophages the ability to phosphorylate STAT-1 and to produce NO as efficiently as C57BL/6 cells. We demonstrate, for the first time, that BALB/c macrophages are innately lower NO producers than C57BL/6 cells because they are defective in the TLR-4-induced IFN-β-mediated STAT-1 activation pathway.

## Introduction

The functions of macrophages as microbicidal, cytokine-producing and antigen-presenting cells make them crucial players in host resistance to a variety of pathogens, in many cases determining a favorable outcome for the infected host. At times, however, macrophages play the reverse role, harboring intracellular microorganisms, thereby providing them appropriate environments for their development and sheltering them from the adaptive immune response, leading to chronic diseases. Whether macrophages kill or house pathogens depends both on the vulnerability of the microorganism and the activation state of the host cells. These can be activated either by pathogens, through their various pathogen-associated molecular patterns (PAMP) [1] or by cytokines [2]. Previous reports have shown that macrophages from Th1- and Th2-prone mice differ in their ability to be activated by the so-called classical activators, IFN-γ and/or LPS. Hence, cells from BALB/c mice (typical Th2 responders) stimulated with LPS not only produce little or no NO, but also increase arginine metabolism of ornithine. In contrast, cells from C57BL/6 mice (typical Th1 responders) produce a strong NO and citrulline response and seem to decrease their production of ornithine [3]. The differential ability of C57BL/6 and BALB/c macrophages to produce NO *in vitro* in response to IFN-γ and LPS or TNF-α had also been demonstrated by several groups, including ours [4]–[11]. C57BL/6 and BALB/c mice are broadly used as prototypes of resistance and susceptibility, respectively, to several infectious diseases. In many cases, resistance is due to the microbicidal effect of NO produced by these cells [12]–[14], particularly in response to IFN-γ produced by Th1 lymphocytes. A well-studied example is the resistance of C57BL/6 to *Leishmania major* infection. Most published work on this model agrees that the resistance of C57BL/6 to *L. major* is dependent of a Th1-induced macrophage NO production or other Th-dependent responses [15], [16]. However, the fact that C57BL/6 macrophages intrinsically produce more NO than BALB/c cells, regardless any ongoing Th response, suggests that macrophage-mediated innate immunity have a more relevant status than currently believed in the intricate mechanisms that confers resistance to the parasite. This led us to start investigating the molecular basis of the differential intrinsic ability of macrophages from C57BL/6 and BALB/c mice to produce NO in response to IFN-γ and LPS. In a previous work [10], we found that the higher production of NO by C57BL/6 macrophages is a consequence of a higher expression of iNOS, which results from higher accumulation of iNOS mRNA, in comparison with BALB/c cells. We also found that in the double-stimulated cells the difference in the accumulation of iNOS mRNA in C57BL/6 and BALB/c macrophages is not a consequence of differential stabilities of the mRNA, suggesting that *iNOS* is transcribed at different rates in C57BL/6 and BALB/c.

In the present study, we sought to identify the differences in the signaling cascade for iNOS synthesis between C57BL/6 and BALB/c macrophages that results in higher NO production by the former, in an attempt to investigate the significance of this difference early in the response in determining the susceptible and the resistant phenotypes in these murine models to some infectious diseases in particular to leishmaniasis. Herein, we stimulated macrophages with LPS without the co-stimulus of IFN- γ to focus on the differences only in TLR4-driven signaling pathways. Our results demonstrated that the difference in the production of NO is not due to a differential availability of L-arginine resulting of a differential arginase activity. Rather, C57BL/6 and BALB/c macrophages differ in the regulation of the expression of iNOS and iNOS mRNA, which appears to reside at the transcriptional level. We found that the surplus of NO in C57BL/6 compared to BALB/c is due to a higher production of IFN-β that leads to a higher activation of STAT-1, indispensable for iNOS transcription.

## Materials and Methods

### Animals and ethics statement

C57BL/6 and BALB/c mice were purchased from Centro de Bioterismo, ICB, UFMG, Belo Horizonte, Brazil. This study was carried out in strict accordance with Brazilian laws governing animal experimentation. The protocol was approved by the Universidade Federal de Minas Gerais Animal Experimentation Ethics Committee (Permit Number 108/2004) and all efforts were made to minimize suffering.

Equipments, consumables and softwares were purchased from: **Brazil**: Cripion Biotecnologia Ltda, Andradina, SP; Life Technologies do Brasil, Vila Guarani, SP. **USA**: BD, San Jose, CA; BD Biosciences, Franklin Lakes, NJ; Bio-Rad laboratories, Inc., Hercules, CA; Cell Signaling Technology Inc., Danvers, MA; Difco, Franklin Lakes, NJ; GE Healthcare, Waukesha, WI; GraphPad Software, Inc., La Jolla, CA; InvivoGen, San Diego, CA; KPL, Gaithersburg, MD; Millipore, Billerica, MA; Molecular Devices, LCC, Sunnyvale, CA; Nalge Nunc International, Rochester, NY; R&D Systems, Minneapolis, MN; Shering-Plough, Whitehouse Station, NJ; Sigma-Aldrich, St. Louis, MO; Tree Star, Inc., Ashland, OR. **The Netherlands**: PBL Biomedical Laboratories, Leiden.

### Cell cultures

Peritoneal macrophages were harvested 4 days after i.p. injection of 2.0 mL of 3% thioglycolate medium (Difco) to 7- to 10-wk-old BALB/c and C57BL/6 mice. Cells were plated in 96- (1×10^5^ cells/well), 24- (3×10^5^ to 1×10^6^ cells/well) or 6-well plates (5×10^6^ cells/well) containing RPMI 1640 (Sigma-Aldrich) supplemented with 10% of heat-inactivated FBS (Cripion Biotecnologia Ltda) and 50 mg/mL of gentamycin (Shering-Plough) (RPMI/FBS) and incubated for 2 h at 37°C and 5% CO_2_. Adherent cells were then washed with RPMI, further incubated overnight under the same conditions, and the medium was replaced by a fresh one. Unless otherwise indicated, cells were cultured in the presence of 1 µg/mL LPS (*Escherichia coli* serotype 0127) (Sigma-Aldrich) or Poly (I:C) (InvivoGen). In some experiments, the supernantant of 8 h-stimulated C57BL/6 cells or 0.5 to 10 U/ml recombinant mouse IFN-β (Millipore) was added to BALB/c cells, which were cultured for the indicated period of time.

Bone marrow macrophages were obtained as described [17]. Briefly, femurs and tibias were flushed with 5 mL of Hank's balanced salt solution (Life technologies do Brasil). The suspension was centrifuged, and cells were resuspended in RPMI/FBS containing 10% L929 cell-conditioned medium (LCCM) as a source of M-CSF [18]. The cells were distributed in 24-well plates and incubated at 37°C and 5% CO_2_. After 3 days, 0.1 mL of LCCM was added and the media was renewed on the seventh day. Cells were used on the 10th day of culture, when completely differentiated into macrophages.

### Quantification of NO

Cell culture supernatants were assayed for NO_2_^−^ accumulation as described [19]. Briefly, 50 µl-samples were incubated with equal volumes of Griess reagent (1% sulphanilamide, 0.1% N-1-naphtylethylenediamine dihydrochloride, 2.5% phosphoric acid) in microplates at room temperature for 10 min. The absorbance at 540 nm was determined in a spectrophotometer (SPECTRAmax 340, Molecular Devices). NO_2_^−^ concentrations were calculated using a standard curve using sodium nitrite.

### RNA isolation and real-time RT-PCR

Total RNA was isolated from macrophage cultures using the Illustra™ RNAspin MiniRNA Isolation Kit, and the reverse transcription was performed using 500 ng of RNA and the IllustraReady-To-Go RT-PCR Beads (GE Healthcare), according to the manufacturer's instructions. Real-Time RT-PCR was conducted in a final volume of 10 µL containing SYBR Green PCR Master Mix (Life Technologies do Brasil), 1 µM of each primer and oligo-dT-cDNA. The PCR reaction was performed with an ABI 7900 Real-Time PCR System (Life Technologies do Brasil) using the following cycling parameters: 60°C, 10 min, 95°C, 10 min, 40 cycles of 95°C, 15 sec and 60°C, 1 min, and a dissociation stage of 95°C, 15 sec, 60°C, 1 min, 95°C, 15 sec, and 60°C, 15 sec. The primers used to amplify the specific fragment corresponding to unique genes were: iNOS - F: CAGCTGGGCTGTACAAACCTT, R: CATTGGAAGTGAAGCGTTTCG; β-actin - F: AGAGGGAAATCGTGCGTGAC, R: CAATAGTGATGACCTGGCCGT [21]; arginase I - F: AAAGCTGGTCTGCTGGAAAA, R: ACAGACCGTGGGTTCTTCAC; arginase II - F: TGGACAGCCACTTTCCTTTC, R: GAGGCTCCACATCTCTCAGG; IFN-β - F: ACGTGGGAGATGTCCTCAACTGC, R: TCGGACCACCATCCAGGCGT; IL-10 - F: TGGACAACATACTGCTAACC, R: GGATCATTTCCGATAAGGCT; TNF-α - F: TGTGCTCAGAGCTTTCAACAA, R: CTTGATGGTGGTGCATGAGA [22]. Data are presented as relative expression units after normalization to β-actin. Reactions were conducted in triplicate.

### Arginase activity

Arginase activity was measured as previously described [23]. Briefly, cells were lysed with 50 µl of 0.1% Triton X-100 (Sigma-Aldrich). The lysate was stirred for 30 min, and 50 µl of 10 mM MnCl_2_ with 50 mM Tris-HCl pH 7.4 were added to activate the enzyme for 10 min at 56°C. Arginine hydrolysis was performed by incubating the lysate with 25 µl of 0.5 M L-arginine (pH 9.7) at 37°C for 60 min. The reaction was stopped by adding 400 µl of a solution containing H_2_SO_4_, H_3_PO_4_ and H_2_O (1∶3∶7). The urea concentration was measured at 540 nm after the addition of 25 µl of α-isonitrosopropiophenone (Sigma-Aldrich) dissolved in ethanol, followed by heating at 95°C for 45 min. The activity of the samples was calculated by using a standard curve generated from known quantities of urea. One unit is defined by the amount of enzyme that catalyzes the formation of 1 µmol of urea per min.

### Protein extracts and western blot analysis

Total cellular proteins were isolated using standard methods [20]. In brief, cell monolayers were washed with PBS and treated with lysis buffer (50 mM Tris–HCl pH 7.4, 150 mM NaCl, 50 mM NaF, 10 mM β-glycerophosphate, 0.1 mM EDTA, 10% glycerol, 1% Triton X-100, 1 mM sodium orthovanadate) supplemented with a protease inhibitor cocktail (GE Healthcare). The lysates were then clarified by centrifugation at 13,000 g for 10 min at 4°C and the protein concentrations of the whole cell lysates were measured by the Bradford protein assay (Bio-Rad laboratories, Inc.). Lysates of the same number of cells (around 40 µg protein) were subjected to electrophoresis on SDS-PAGE gels followed by Western blot according to standard techniques, using the following monoclonal antibodies: anti-Nitric Oxide Synthase, Inducible (Sigma-Aldrich), anti-phospho(p)-NF-κB p65 (Ser536), anti-IκB-α, anti-STAT-1, anti-p-STAT-1 (Y701) and anti-β-actin (Cell Signaling Technology Inc.). Peroxidase-labeled anti-Rabbit IgG (KPL) was used as the secondary antibody. Immunoreactive bands were visualized using a Luminol chemiluminescent HRP substrate (Millipore) and were analyzed in a Storm System 860 (GE Healthcare). Densitometry analyses were performed using ImageJ 1.44p (National Institutes of Health, Bethesda, MD).

### iNOS protein degradation

Macrophage cultures were incubated with LPS (1 µg/mL) for 15 h and exposed to 50 µg/mL cycloheximide (CHX) to stop protein synthesis. Cell monolayers were washed with PBS at 0, 1, 3, 5 and 7 h after the addition of CHX, and the total cellular extracts was used in Western blot analysis to detect iNOS. As a control of translational inhibition, the cell monolayers were exposed to CHX for 2 h before LPS stimulation.

### Neutralization of IFN-β, IL-10 and TNF-α

The macrophages were incubated with LPS (1 µg/mL) or supernantants of LPS-stimulated cells for 8 to 72 h in the presence of one of the following neutralizing antibodies: rabbit polyclonal antibody specific for IFN-β (PBL Biomedical Laboratories), mouse monoclonal IgG antibody specific for IL-10, kindly donated by Dr. Milton Adriano Pelli de Oliveira (Universidade Federal de Goiás, Brazil), rat monoclonal IgG antibody specific for IL-10 receptor (Harlan Bioproducts Science) (kindly donated by Dr. David Sacks, NIH, USA), and mouse-human chimeric anti-human TNF-α antibody (Infliximab) (Remicade, Janssen Biotech Inc.), known to recognize mouse TNF-α, a gift from Dr. Aristóbolo Mendes da Silva, UFMG, Brazil). Supernatants were then collected for NO quantification, and total cellular protein was extracted for Western blot analysis. Normal rabbit IgG was used as isotype control.

### Cytokines measurement

IL-10 and TNF-α were measured in macrophage supernatants by ELISA using the mouse IL-10 or the mouse TNF (Mono/Mono) ELISA Set, respectively (R&D Systems), according to the manufacturer's instructions.

### Data representation

The results of this study were represented as a typical experiment of at least three independent and reproducible experiments, which are shown as supplemental figures.

## Results

### Differential sensitivity of C57BL/6 and BALB/c macrophages to LPS NO production

**Figure 1:**
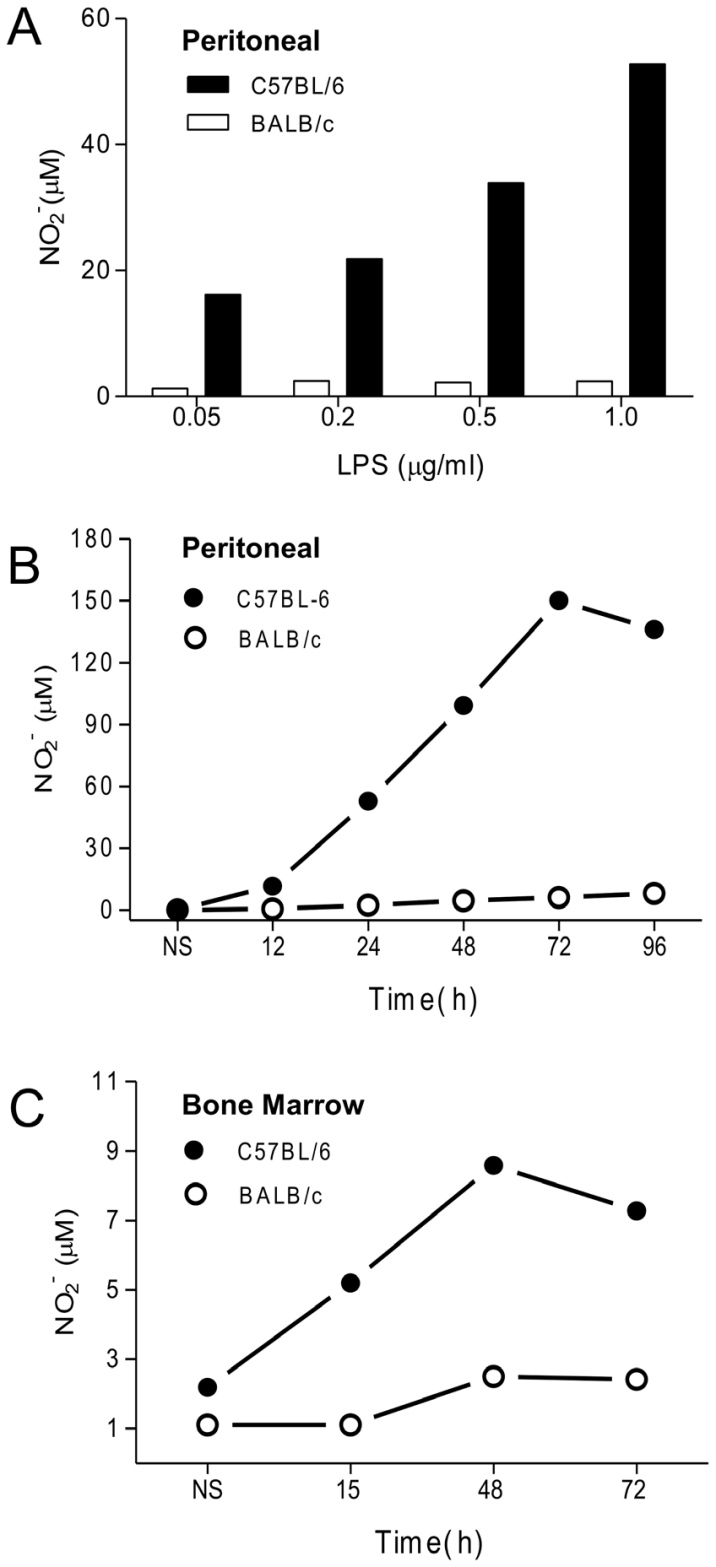
NO production by BALB/c and C57BL/6 macrophages. Peritoneal or bone marrow macrophages (1×10^5^) were stimulated with LPS at the indicated concentrations for 72 h (A) or with 1 µg/mL LPS for the indicated periods of time (B and C). Culture supernatants were analyzed for NO^−^_2_ concentration using the Griess reaction, as described in the Materials and Methods. Values correspond to the mean of triplicate wells, and data are representative of three independent and reproducible experiments. Additional experiments to illustrate the variability in the results are shown in Fig. S1.

Previous results have shown that C57BL/6 and BALB/c macrophages are differentially sensitive to LPS plus IFN-γ for the production of NO [10]. Here, in an attempt to identify the differences in the TLR4-induced signaling cascade for NO production, we used LPS only. We initially show a dose-response and a time-course of NO production in response to LPS. We can observe the striking difference in NO production between C57BL/6 and BALB/c macrophages and the concentration-dependence of C57BL/6 cells response (Fig. 1A). At the concentration of 1 µg/mL LPS, which induced the maximal difference between the two mice strains, the increase in NO production over time is dramatic in C57BL/6 and almost negligible in BALB/c. The difference remarkably increases during 72–96 h, when is maximal (Fig. 1B). Bone marrow-derived macrophages from C57BL/6 and BALB/c are also differentially sensitive to LPS, behaving like the thioglycolate-elicited cells (Fig. 1C). In different experiments, we may see a variation in the amount of NO produced, but in all of them there is always at least 3 times more NO produced by C57BL/6 macrophages at this concentration of LPS. Cell viability is >95% up to 72 h, when it decreases to approximately 30% (data not shown), so that the amount of NO measured at 72–96 h reflects the accumulated nitrite.

### Differential production of NO by C57BL/6 and BALB/c macrophages is not due to a difference in arginase activity

**Figure 2:**
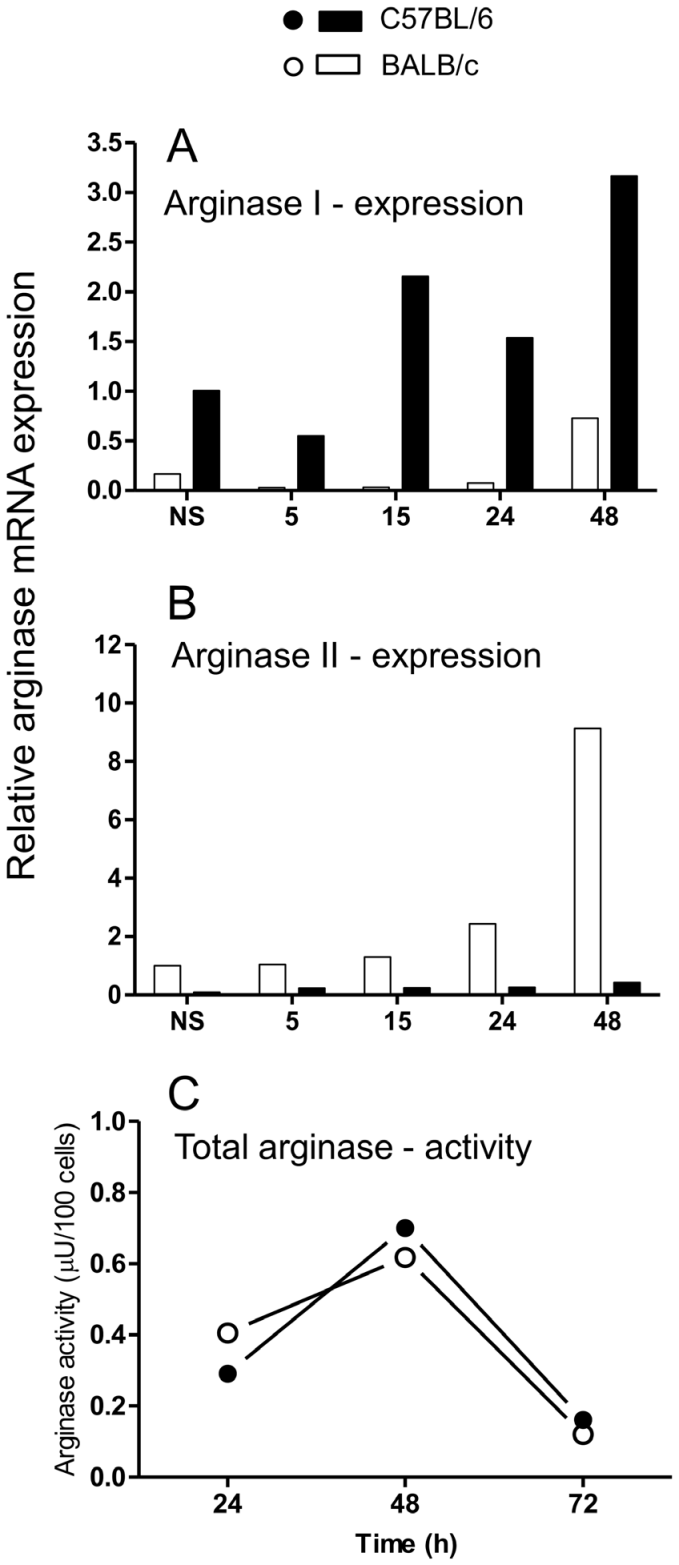
Arginase expression and activity in BALB/c and C57BL/6 macrophages. Cells (5×10^6^) were stimulated with 1 µg/mL LPS for the indicated periods of time. Total RNA was extracted, and mRNA levels of arginase I (A) and arginase II (B) were determined by Real Time RT-PCR. The relative levels of gene expression were calculated by reference to the β-actin expression in each sample, using the 2^−ΔΔCt^ method. Values represent the mean of samples assayed in triplicate. After stimulation with 1 µg/mL LPS for the indicated time, peritoneal macrophages (5×10^5^) were washed, lysed and assayed for arginase activity by urea quantification (C). Values correspond to the difference between stimulated and non-stimulated cells, and the data are representative of three independent and reproducible experiments. Additional experiments to illustrate the variability in the results are shown in Fig. S2.

In a previous report [10], we had not ruled out the participation of arginase in the differential production of NO by C57BL/6 and BALB/c macrophages. In order to verify whether the lower production of NO by BALB/c macrophages was due to a depletion of l-arginine by arginase, we investigated the expression and the activity of this enzyme. Notably, the constitutive expression of arginase I is higher in C57BL/6 than in BALB/c macrophages (Fig. 2A) and the opposite is true for arginase II (Fig. 2B). Moreover, LPS preferentially induces the upregulation of arginase I and II in C57BL/6 and BALB/c macrophages, respectively. However, the activity of total arginase after stimulation with LPS increased similarly in both types of macrophages, following a kinetic that matches mRNA expression (Fig. 2C). By 72 h, arginase activity decreased to similar levels in the macrophages. This result rules out the contribution of arginase to the differential production of NO by C57BL/6 and BALB/c macrophages in response to LPS.

### Differential production of NO in C57BL/6 and BALB/c macrophages correlates with the expression of the enzyme iNOS

**Figure 3:**
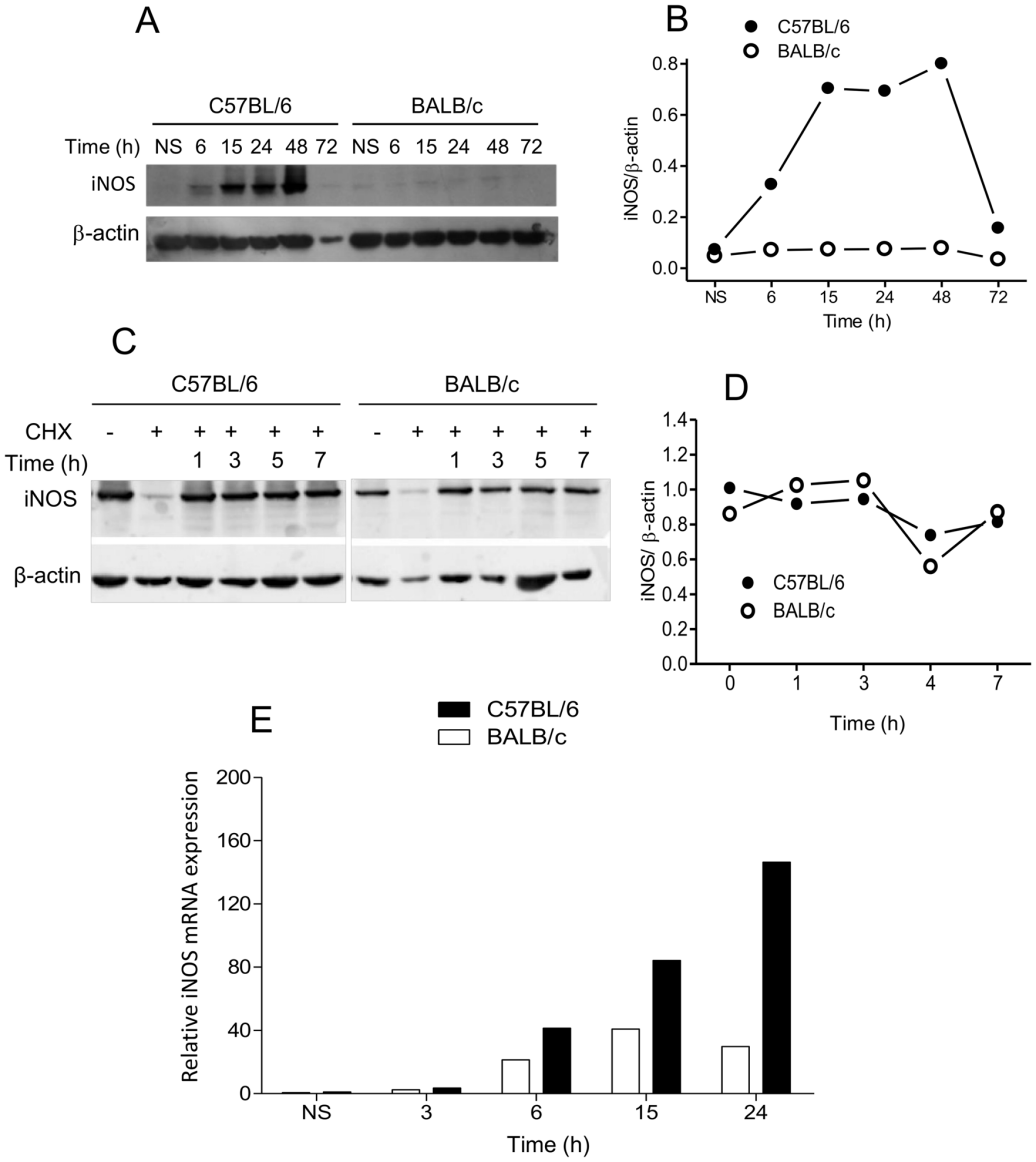
iNOS protein and mRNA expression in BALB/c and C57BL/6 macrophages. Cells (1×10^6^) were stimulated with 1 µg/mL LPS for the indicated periods of time (A and B) or for 15 h and treatment with 50 µg/mL cycloheximide for the indicated periods of time (C and D). “**+**” ou “**−**” only represents cells that were previously treated with cycloheximide for 2 h before LPS treatment. Total protein extracts were analyzed by Western blot using specific anti-iNOS and anti-β-actin antibodies. The levels of iNOS were expressed as ratios of the signal intensity of the bands normalized to that of β-actin (B and D). Cells (5×10^6^) were stimulated with 1 µg/mL LPS for the indicated periods of time. Total RNA was extracted, and mRNA levels of iNOS were determined by Real Time RT-PCR. The relative levels of iNOS mRNA were calculated by reference to the β-actin expression in each sample, using the 2^−ΔΔCt^ method (E). Values correspond to the mean of samples assayed in triplicate. Data are representative of three independent and reproducible experiments. Additional experiment to illustrate the variability in the result is shown in Fig. S3.

We next investigated whether the LPS-induced differential production of NO by C57BL/6 and BALB/c macrophages was due to differences in the levels of iNOS. We showed by Western blot that LPS induces a strong expression of iNOS (130 kDa) (Fig. 3A) in C57BL/6 macrophages, detected as early as 6 h (Fig. 3B). The production of iNOS in C57BL/6 macrophages continues to increase over the next 42 h, and by 72 h it is again undetectable, probably due to the low cell metabolism. At the peak points, the expression of iNOS in C57BL/6 macrophages is, depending on the experiment, 3-7-fold higher than that of BALB/c macrophages, which barely express the protein (Fig. 3B) and never reach comparable levels to C57BL/6 macrophages over the whole period of time.

### Differential accumulation of iNOS in C57BL/6 and BALB/c macrophages is not a result of protein differential degradation and correlates with the expression of iNOS mRNA

The higher accumulation of iNOS in C57BL/6 macrophages could be a consequence of a lower degradation rate of iNOS, a higher accumulation of its mRNA, or both. Using Western blot, we saw that the enzyme is equally stable in C57BL/6 and BALB/c macrophages for 7 h (then macrophages die) in the presence of cycloheximide at a non- toxic concentration that inhibits translation (Fig. 3C and D). This indicates that steady-state iNOS abundance in C57BL/6 macrophages and scarcity in BALB/c cells is not controlled by mechanisms affecting protein stability, suggesting that the difference in iNOS production is a consequence of differential mRNA accumulation. Indeed, real-time PCR showed that C57BL/6 and BALB/c macrophages differ markedly in the expression of iNOS mRNA. It is observed as early as 3 h in both types (Fig. 3E) but, starting at 6 h, the difference between the two clearly increases. BALB/c macrophages accumulate a relatively low level of iNOS mRNA up to 15 h, decreasing thereafter, whereas in C57BL/6 cells, mRNA levels continue to increase up to 24 h, when they are approximately 5-7-fold higher (depending on the experiment) than in BALB/c (Fig. 3E). Since the half-life of iNOS mRNA is the same in both types of macrophages [10], these results, together, suggest that C57BL/6 and BALB/c macrophages differently regulate the transcription of iNOS following TLR4 stimulation.

### Expression macrophages iNOS mRNA correlates with STAT-1, but not NF-κB, activation

**Figure 4:**
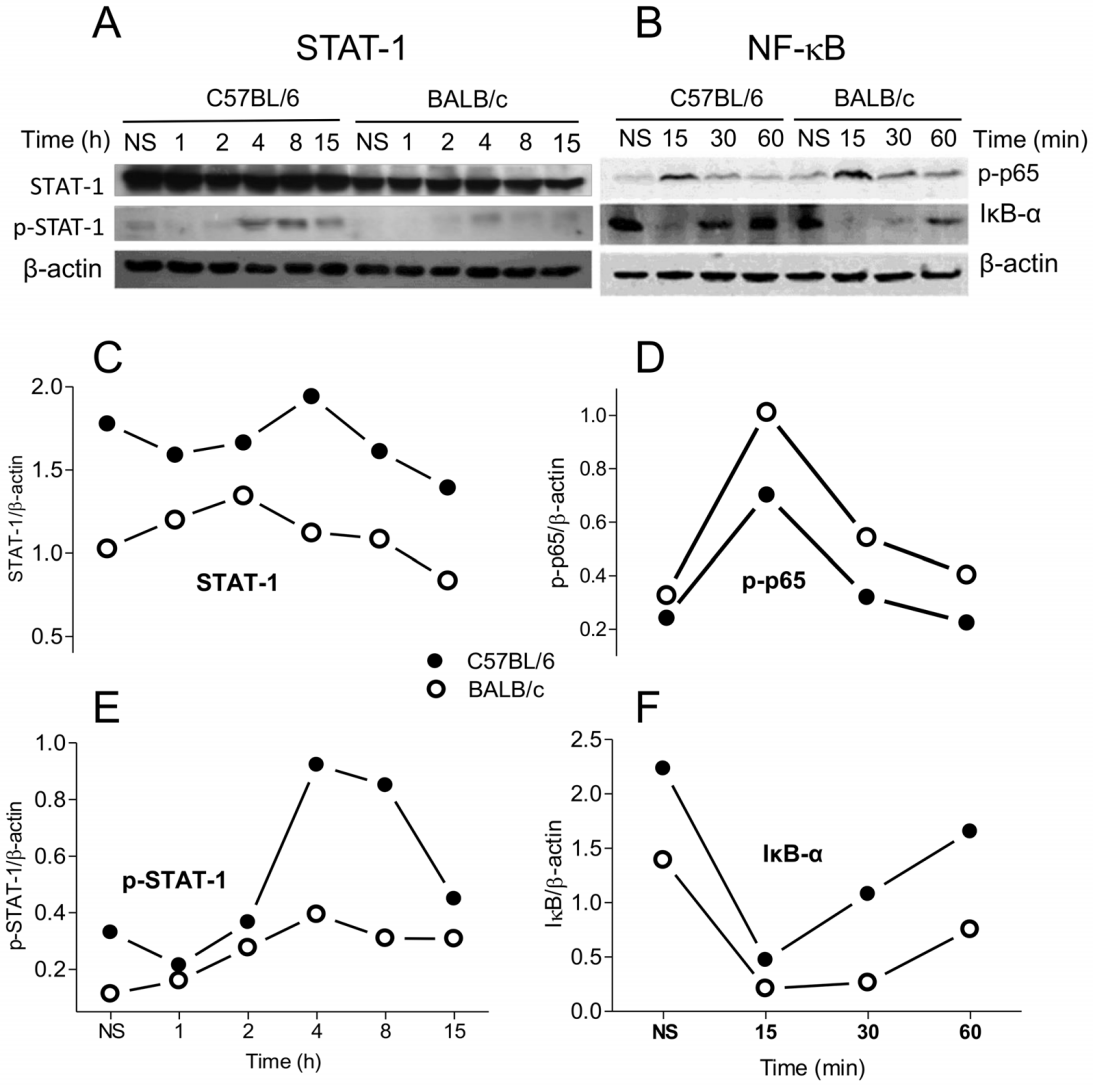
STAT-1 and NF-κB expression and activation in BALB/c and C57BL/6 macrophages. Total protein extracts of C57BL/6 or BALB/c peritoneal macrophages (1×10^6^) stimulated with 1 µg/mL LPS for the indicated periods of time were analyzed by Western blot using specific anti-STAT-1, anti-pSTAT-1 (Y701) (A) or anti-pp65 (Ser536), anti-IκB-α (B) and anti-β-actin antibodies (A and B). The levels of STAT-1 (C), p-STAT-1 (E), p-p65 (D) or IκB-α (F) were expressed as ratios of the signal intensity of the bands normalized to that of β-actin. Data are representative of three independent and reproducible experiments.

**Figure 5:**
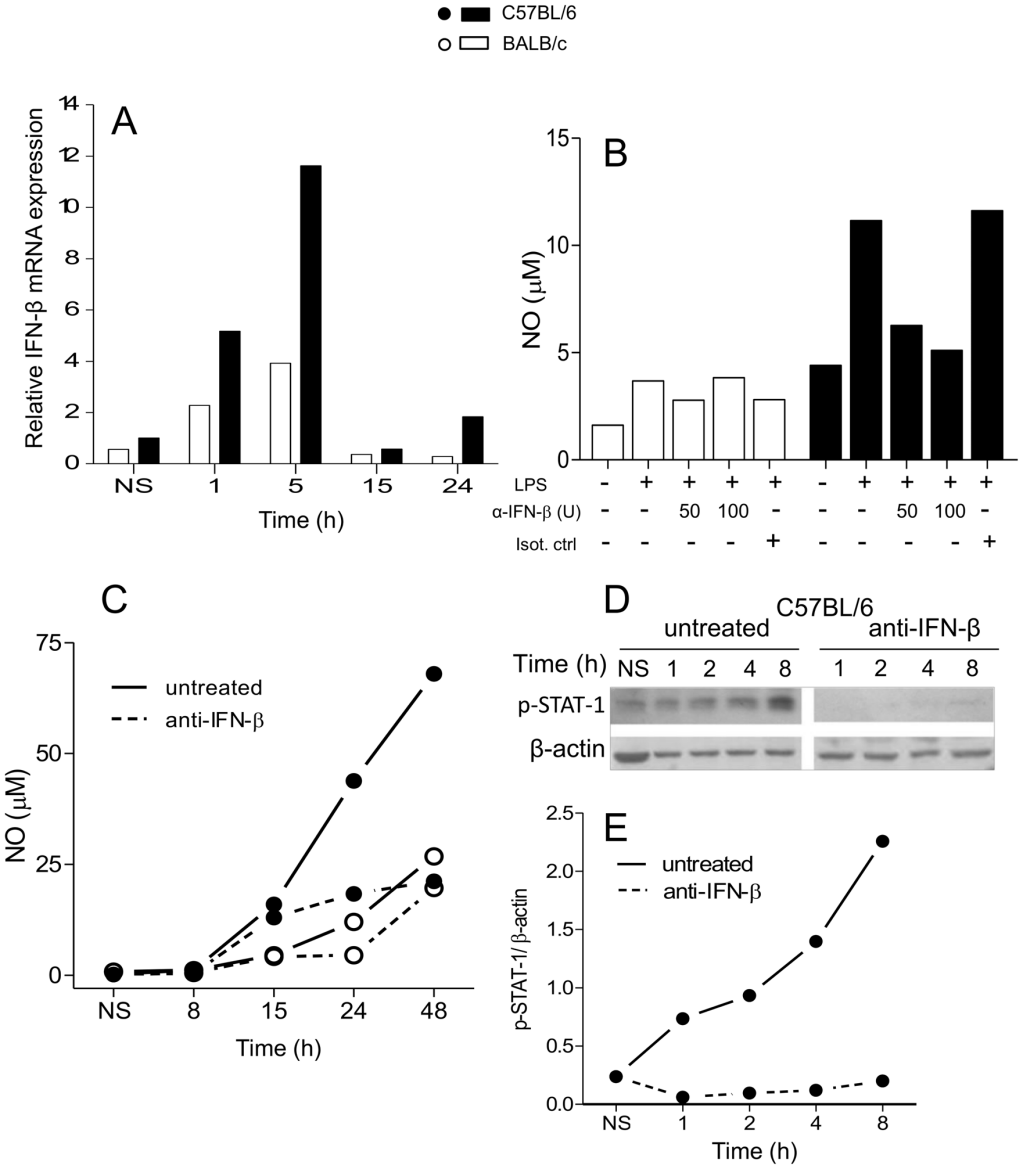
IFN-β mRNA expression and effect of IFN-β neutralization on NO production and STAT-1 activation in BALB/c and C57BL/6 macrophages. Peritoneal macrophages (5×10^6^) were stimulated with 1 µg/mL LPS for the indicated periods of time. Total RNA was extracted and IFN-β mRNA levels were determined by Real Time RT-PCR. The relative levels of mRNA expression were calculated by reference to the β-actin expression in each sample, using the 2^−ΔΔCt^ method (A). Alternatively, cells (1×10^5^) were stimulated for 24 h (B) or for the indicated periods of time (C–E) with 1 µg/mL LPS in the presence or absence of the indicated concentrations (B) or with 200 U/mL of polyclonal anti-IFN-β (C–E). Culture supernatants were analyzed for NO^−^_2_ levels using the Griess reaction, as described in the Materials and Methods. Purified normal rabbit IgG was used as an isotype control. (B) represents the dose-response of one experiment and (C) represents the time course of a second one, out of three independent experiments. Values represent the mean of samples assayed in triplicate. (D and E) represents STAT-1 activation as analyzed by Western blot of total protein extracts of C57BL/6 peritoneal macrophages (3×10^5^) using antibodies specific for pSTAT-1 (Y701) and β-actin. The levels of pSTAT-1 (E) were expressed as ratios of the signal intensity of the bands normalized to that of β-actin. Data are representative of three independent and reproducible experiments. Additional experiments to illustrate the variability in the results are shown in Fig. S4.

**Figure 6:**
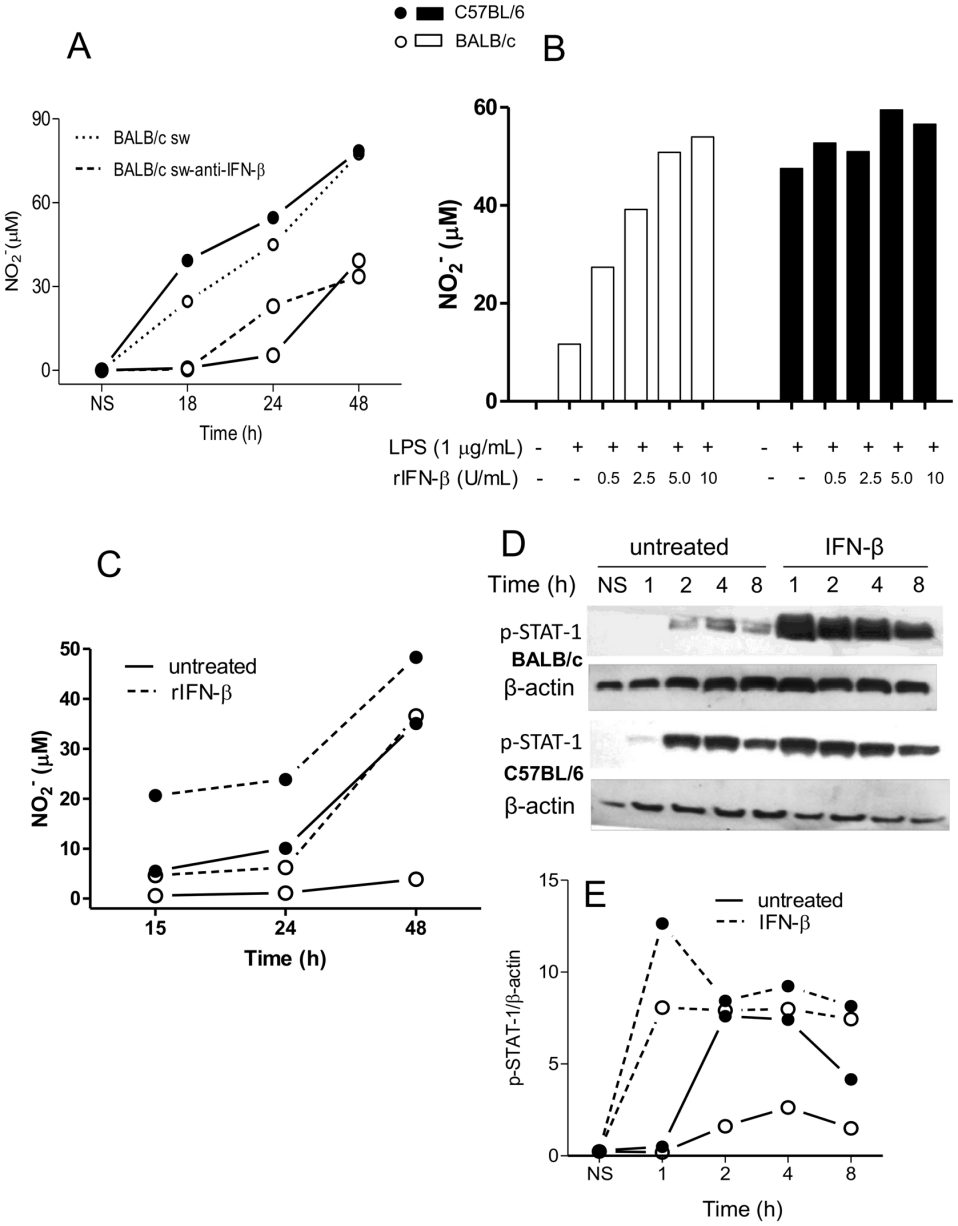
Effect of exogenous IFN-β on NO production and STAT-1 activation in BALB/c and C57BL/6 macrophages. Peritoneal macrophages (1×10^6^) were stimulated with 1 µg/mL LPS for the indicated periods of time. After 8 hs, supernatants of C57BL/6 and BALB/c cells, still devoid of NO, were swapped and, cells were cultured in the presence (dotted lines) or absence (dashed lines) of anti- IFN-β for the indicated periods of time, when supernatants were analyzed for NO^−^_2_ levels using the Griess reaction, as described in the Materials and Methods (A). Alternatively, 1×10^6^ (B and C) or 3×10^6^ (D and E) were stimulated for 48 h (B) or for the indicated periods of time (C–E) with 1 µg/mL LPS in the presence or absence of the indicated concentrations (B) or with 10 U/mL rIFN-β (C–E). Culture supernatants were analyzed for NO^−^_2_ levels as above (B and C). (B) represents the dose-response of one experiment and (C) represents the time course of a second one, out of three independent experiments. Values represent the mean of samples assayed in triplicate. D and E represent STAT-1 activation as analyzed by Western blot of total protein extracts of C57BL/6 peritoneal macrophages (3×10^5^) using antibodies specific for pSTAT-1 (Y701) and β-actin. The levels of pSTAT-1 (F) were expressed as ratios of the signal intensity of the bands normalized to that of β-actin. Data are representative of three independent and reproducible experiments. Additional experiments to illustrate the variability in the results are shown in Fig. S5.

STAT-1 and NF-κB are key trans-acting factors involved in the transcription of iNOS [24]. In an attempt to determine whether these transcription factors are differentially expressed and/or activated in C57BL/6 and BALB/c macrophages stimulated with LPS, we carried out Western blot experiments using antibodies specific for STAT-1, p-STAT-1, p-NF-κB p65 and its inhibitor, IκB-α. We observed that the levels of total STAT-1 is higher in C57BL/6 than in BALB/c macrophages (Fig. 4A and C). We also saw that a doublet band (best seen in Figs. 5C and 6) reacted with the anti-p-STAT-1 antibody (Fig. 4A) that may correspond to p91 (STAT-1-α) and p84 (STAT-1-β) [25]. Thioglycolate-elicited C57BL/6, but not BALB/c macrophages, already has some phosphorylated STAT-1 that tends to disappear within 1 hour after harvesting (Fig. 4A). While LPS-induced phosphorylation of STAT-1 is noticeable in BALB/c macrophages, it is clearly stronger and longer lasting in C57BL/6 (Fig. 4A). Quantification of the two bands shows an approximate 2-3-fold difference in the levels of p-STAT-1 at 4 and 8 h, respectively, in C57BL/6 and BALB/c macrophages (Fig. 4E). As to NF-κB, p65 is phosphorylated in macrophages of both strains of mice by 15 min after LPS induction (Fig. 4B), although a somewhat greater amount of p65 seems to be phosphorylated in BALB/c macrophages, based on its expression in Western blot (Fig. 4D). More evidently, NF-κB is active for a longer period of time in BALB/c than in C57BL/6 macrophages, as judged by the appearance of IκB-α in the cytosol at 30 and 60 min (Fig. 4B and F). These data do not correlate with the higher expression of iNOS in C57BL/6 macrophages. Together, these results indicate that STAT-1, but not NF-κB, may account for the higher expression of iNOS in C57BL/6 macrophages.

### IFN-β is responsible for the differential STAT-1 activation and NO production by C57BL/6 and BALB/c macrophages

Because the binding of LPS to TLR4 does not directly induce STAT-1 activation [26], [27], we searched for cytokines that do perform this function. LPS induces the expression of TNF-α, IFN-β, IL-1β, IL-6, IL-10 and IL-12 in murine peritoneal macrophages [28], among which IFN-β is known to induce the phosphorylation of STAT-1 and to contribute to iNOS expression [29], [30]. Therefore, we investigated the expression of IFN-β in LPS-stimulated C57BL/6 and BALB/c macrophages. Figure 5A shows that, indeed, thioglycolate-elicited C57BL/6 cells consistently produced about twice as much IFN-β mRNA than BALB/c. When stimulated by LPS, C57BL/6 and BALB/c cells further differentially upregulate the expression of IFN-β, which peaks at 5 h in both cell types. In this experiment, the relative amount of IFN-β mRNA in C57BL/6 macrophages was approximately 2.5- and 3-fold (up to 10-fold in other experiments) higher than in BALB/c cells by 1 and 5 h, respectively. At 15 h, both cell types have returned to basal levels, but at 24 h, a smaller second wave of IFN-β expression is detectable in C57BL/6 macrophages.

To investigate whether IFN-β was being secreted and subsequently stimulating the production of NO, an anti-IFN-β neutralizing antibody was used. Figure 5B depicts that the treatment of LPS-stimulated macrophages with anti-IFN-β blocked the production of NO by C57BL/6, but not by BALB/c cells, in a dose-dependent manner, and figure 5C represents the whole time-course of another experiment. Normal rabbit IgG does not interfere with the production of NO by mouse macrophages (Fig. 5B). It is clear that upon neutralization of IFN-β, C57BL/6 macrophages produce as little NO as BALB/c cells. This demonstrates that the differential NO production resides in the differential ability of the macrophages to synthesize IFN-β. Neutralization of IFN-β does not eliminate the production of NO in either C57BL/6 or BALB/c macrophages, indicating that other LPS-induced pathways of iNOS synthesis that are independent of IFN-β are functioning and comparable in both types of cells. The neutralization of IFN-β in C57BL/6 cells also abolishes the phosphorylation of STAT-1, which is essential for the full transcription of iNOS (Fig. 5D and E). A counterproof experiment shows that supernatants from 8-h-LPS-stimulated C57BL/6 macrophages (still devoid of NO) are able to make BALB/c cells as high NO-producers as those of C57BL/6. Neutralization of IFN-β prevents the cells to produce NO, corroborating that IFN-β is the responsible for inducing the additional NO production in C57BL/6 macrophages (Fig. 6A). Addition of rIFN-β to LPS-stimulated BALB/c cells recovers, in a dose-dependent manner, their potential to produce as much NO as C57BL/6 cells, demonstrating that what lacks in BALB/c mice macrophages to produce a high NO response is IFN-β (Fig. 6B). Figure 6C shows the time-course of another similar experiment. Crucially, rIFN-β also induces in LPS-stimulated BALB/c macrophages as much phosphorylation of STAT-1 as in C57BL/6 cells (Fig. 6D and E). Since STAT-1 is crucial for iNOS transcription, this shows that the surplus NO produced by C57BL/6 cells is due to the IFN-β-mediated activation of STAT-1, thereby increasing iNOS transcription. Poly (I:C), a TLR3 ligand known to induce the synthesis of IFN-β, also induced a differential production of NO by peritoneal macrophages and bone-marrow derived macrophages (not shown), corroborating the above results.

### TNF-α and IL-10 are not involved in the differential production of NO

**Figure 7:**
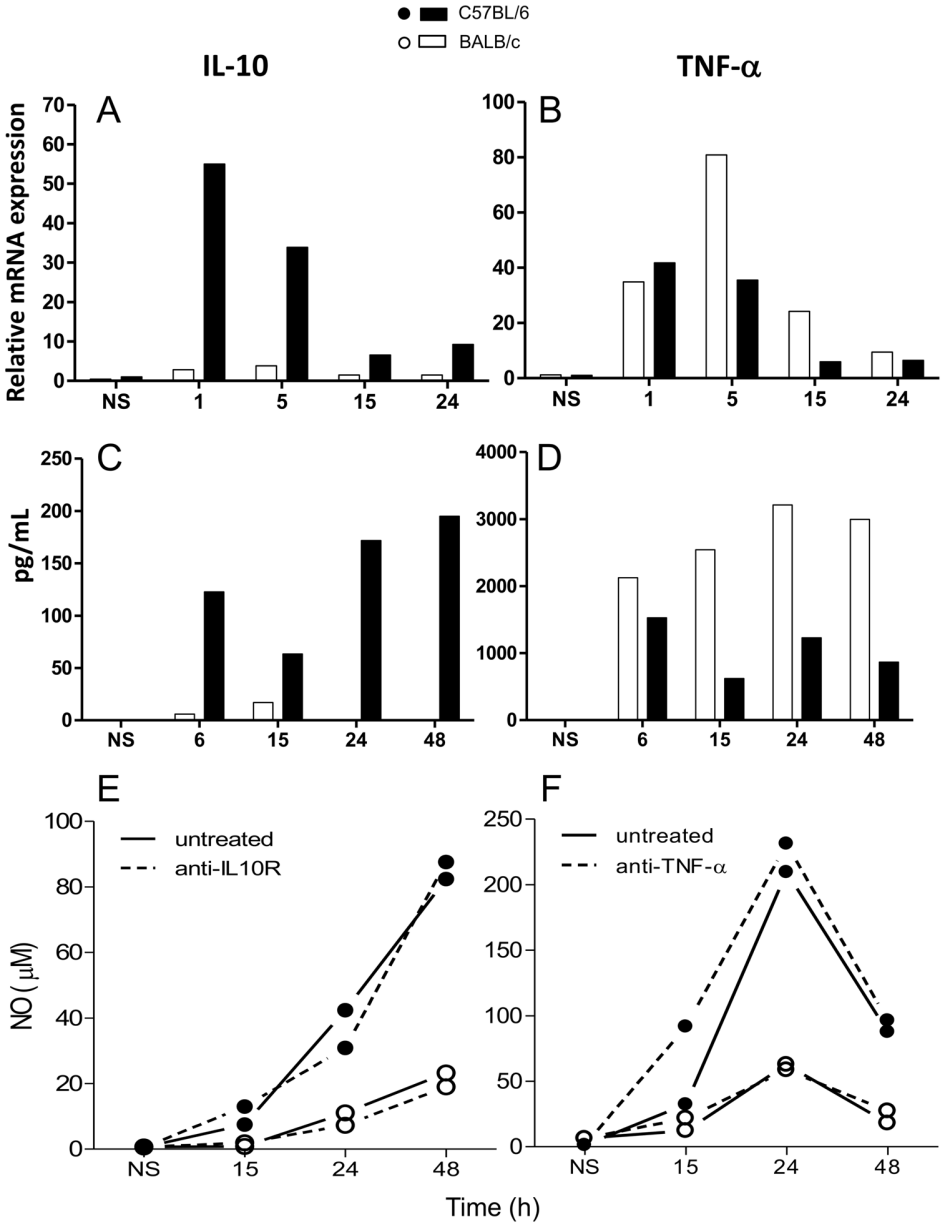
IL-10 and TNF-α expression and effect of IL-10 or TNF-α neutralization on NO production in BALB/c and C57BL/6 macrophages. Cells (1×10^6^ or 5×10^6^) were stimulated with 1 µg/mL LPS for the indicated periods of time. Total RNA was extracted and IL-10 (A) and TNF-α (B) mRNA levels were determined by Real Time RT-PCR. The relative levels of mRNA expression were calculated by reference to the β-actin expression in each sample, using the 2^−ΔΔCt^ method. Protein levels of IL-10 (C) or TNF-α (D) were measured in culture supernatants by ELISA. Values represent the mean ±SD of samples assayed in triplicate. Alternatively, cells (1×10^5^) were stimulated for indicated periods of time (E and DR) with 1 µg/mL LPS in the presence or absence of 10 µg/mL of either a rat monoclonal IgG antibody specific for IL-10 receptor (E) or a mouse-human chimeric anti-human TNF-α antibody (Infliximab) (F). Culture supernatants were analyzed for NO^−^_2_ levels using the Griess reaction, as described in the Materials and Methods. Values represent the mean of samples assayed in triplicate. Data are representative of three independent and reproducible experiments. Additional experiments to illustrate the variability in the results are shown in Fig. S6.

We also examined the expression of IL-10 and TNF-α, which usually inhibits and induces, respectively, NO production. We show that C57BL/6 cells expressed more IL-10 and less TNF-α than BALB/c, both at the mRNA (Figs. 7A and B) and protein (Figs. 7C and D) levels. Thus, expression of IL-10, but not TNF-α, correlates with the synthesis of iNOS. Neutralization of IL-10 or TNF-α does not significantly affect (Fig. 7E and F), or only delayed (Fig. S6 E and F), NO synthesis by C57BL/6 or BALB/c macrophages (Fig. 7E and F). This shows that IL-10 and TNF-α are not involved in the differential production of NO.

## Discussion

NO is a major effector molecule of macrophage antimicrobial and anti-tumor machinery [31]. The enzyme that catalyzes NO synthesis, iNOS, is induced by a variety of cytokines [32] and PAMP ligands of TLRs [33]–[37]. Even though a large amount of information regarding immune responses in infectious diseases has been produced, better understandings on the host resistance mechanisms are still needed. The finding that T helper lymphocytes differ in their patterns of cytokine production [38] led to a breakthrough in the understanding of host resistance to infectious diseases. In the model of *Leishmania major* infection, it has been shown that C57BL/6 mice, a healer phenotype, polarize their response to Th1 whereas the non-healer BALB/c mice polarize it to Th2 [39], [40]. Also in this model, unequivocal evidence showed that NO produced by macrophages activated by Th1-derived IFN-γ is the main component in parasite control/eradication [41], [42].

The present work added a novel piece of information regarding the role of innate immunity by showing why C57BL/6 mice macrophages are much more responsive to TLR4-stimulated NO production than BALB/c cells, regardless of the adaptive response. Our interest in this differential response resides in the fact that, *in vivo*, early parasite containment, which is T cell-independent, NK cell- and IFN-γ-dependent [43], and mediated by IFN-α/β-induced iNOS [44], is crucial for resistance of C57BL/6 to *L. major* [43]. Our assumption is that, before C57BL/6 mice can produce a Th1 response, the inherent ability of their macrophages to produce adequate levels of NO, in response to PAMP or cytokines, would limit pathogen growth, providing additional advantage to the Th1 responders. Conversely, the low ability of BALB/c cells to produce NO would favor microbe growth, aggravated by a Th2 response.

Our results that C57BL/6 macrophages are considerably more responsive to LPS than BALB/c cells in producing NO agree with several reports using IFN-γ plus TNF-α or LPS [3], [6]–[10]. However, the molecular basis for the differential regulation of *iNOS* in C57BL/6 and BALB/c macrophages remains unknown. Isolating the TLR4-mediated signaling, by using LPS only, we showed that the low NO production by BALB/c cells are not due to a delay in its synthesis (Fig. 1) or to a depletion of L-arginine by arginase (Fig. 2C). The higher NO synthesis by C57BL/6 cells correlates with the higher accumulation of iNOS (Fig. 3A and B), which, in turn, correlates with a higher iNOS mRNA expression (Fig. 3E). These accumulations are not due to a higher protein (Fig. 3C and D) or mRNA [10] stability. Like in LPS/IFN-γ-double stimulated cells [10], the sole ligation of TLR4 differentially regulates iNOS expression in C57BL/6 and BALB/c macrophages. Together with the facts that the double-activated C57BL/6 macrophages express more p-STAT-1 and IRF-1, and that STAT-1 remains activated for longer than in BALB/c cells (unpublished results), these results strongly suggest that transcription of iNOS is more efficient in C57BL/6 than in BALB/c macrophages. This does not rule out that differences in the translation rate may also occur, as we previously suggested for LPS/IFN-γ-stimulated C57BL/6 and BALB/c macrophages [10].

In LPS-stimulated macrophages, although STAT-1 and NF-κB are activated in both strains of mice, the degree or the timing of activation is different between them (Fig. 4). STAT-1 phosphorylation is not only stronger in C57BL/6, but also lasts longer than in BALB/c macrophages (Fig. 4A and E), whereas NF-κB activation in BALB/c cells is somewhat more prominent and lasts longer than in C57BL/6 cells (Fig. 4B, D and F). It is possible that the amount of active NF-κB in C57BL/6 macrophages, where STAT-1 is effectively activated, is sufficient for the full transcription of iNOS, while the scarcity of p-STAT-1 in BALB/c macrophages (Fig. 4A and E) impairs transcription of *iNOS* even in an environment of a stronger NF-κB activation. This result confirms that NF-κB is not sufficient for the transcription of *iNOS* and points STAT-1 as a candidate accountable for the differential expression of iNOS by the two types of macrophages.

It has long been known that endogenous IFN-β provides an essential signal for LPS-triggered NO production by murine macrophages [30], [45]. Moreover, the expression of iNOS in macrophages activated by the sole stimulation of TLR4 is dependent on the phosphorylation of STAT-1, which is largely attributed to this cytokine [45], [46], which is confirmed here (Figs. 5 and 6). The novelties are: 1) this important pathway for iNOS transcription is severely impaired in BALB/c macrophages; 2) this is the reason why these cells are unable to produce as much NO as C57BL/6 macrophages. The latter conclusion is clearly demonstrated by the combined facts that 1) neutralization of IFN-β a) abolishes the phosphorylation of STAT-1 in LPS-stimulated C57BL/6 macrophages (Fig. 5D and E), b) leaves them equivalent to BALB/c cells as to the production of NO (Figs. 5B and C), and c) abrogates the ability of LPS-stimulated C57BL/6 macrophages supernatants to induce in BALB/c cells a NO production equivalent to that of C57BL/6 (Fig. 6A) and that 2) exogenous rIFN-β induces the STAT-1 phosphorylation (Fig. 6D and E) and, consequently, the production of NO in BALB/c macrophages (Fig. 6B and C). In the light of the well-established knowledge that the binding of p-STAT-1 to iNOS promoter is essential for the full transcription of the gene [24], [30] and of our results showing that LPS-treated BALB/c macrophages phosphorylates very little STAT-1 in comparison to LPS-treated C57BL/6 (Fig. 4), which is restored by rIFN-β (Fig. 6D and E), it is predictable that, in a context of no p-STAT-1, no binding to iNOS promoter will occur and full transcription of iNOS cannot take place, curtailing NO production, as in LPS-treated C57BL/6 lacking IFN-β (Fig. 5D and E). The restoration of NO production by rIFN- β BALB/c macrophages also reveals that BALB/c deficiency lies in the TLR4 rather than in the IFN- β signaling, which we are currently investigating. BALB/c macrophages are also defective in the TLR3-mediated signaling for NO production, since they are also poorly stimulated by poly (I:C) as compared to C57BL/6 cells (not shown). The fact that poly (I:C) induces IFN-β production [34] through TRIF/IRF-3 pathway further reinforces that the impairment to produce IFN-β is a key determinant in the deficiency of BALB/c macrophages in producing NO and suggests that the deficiency resides in this branch of TLR4-signaling. Because NF-κB activation (Fig. 4) and TNF-α expression (Fig. 7) do not correlate with iNOS expression, ligation of TLR4 in C57BL/6 macrophages seems indeed to signal preferentially through Myd88- PKR-independent/TRIF-dependent pathways, involved in IFN-β production, through IRF-3 activation [47], as opposed to BALB/c cells. Thus, in C57BL/6 and BALB/c macrophages, TLR4 signals toward the autocrine activations of IFN-β and TNF-α, respectively.

IL-10 and TNF-α are usually known to, respectively, inhibit [48] or induce [49] NO synthesis. In LPS-activated macrophages, however, the differential productions of IL-10 (Fig. 7A and C) and TNF-α (Fig. 7B and D) do not account for the differential iNOS expression since their neutralization does not impair NO synthesis by C57BL/6 cells to the level of BALB/c cells or vice-versa (Fig. 7E and F). This is coherent with the inverse correlation of NF-κB activation or TNF-α production with iNOS expression (Figs. 3, 4, 7B and D). The facts that TNF-α is less expressed in C57BL/6 than in BALB/c cells, and the opposite is observed for IL-10 and IFN-β (Figs. 5 and 7), are also consistent with the activation of the transcription factors involved in the expression of *TNF-α* (NF-κB) [50], *IL-10* [51] and *IFN-β* (STAT-1) [48].

The involvement of NO [52], IFN-β [53] and TLR4 [54], [55] in early resistance to *L. major* has already been established in mice. Moreover, *Leishmania* proteoglycolipids [55] and glycoinositol phospholipids [56] are stimulators of macrophage TLR4, and C57BL/6 and BALB/c macrophages diffentially produces NO when stimulated by *Leishmania* glycoinositol phospholipids [56] or lipophosphoglycans [57]. *In vitro*, *L. major* triggers the release of IFN-α/β by macrophages, and neither one alone induces iNOS. Furthermore, IFN-α/β-positive macrophages are detected in the skin of C57BL/6 mice at day 1 of infection [43]. No comparative studies have determined, so far, why the TLR4-induced NO synthesis in macrophages is higher in C57BL/6 than in BALB/c, without Th cells interference. Here we show, for the first time, that macrophages from BALB/c mice, which die from *L. major* infection, are intrinsically low NO producers because they are defective in the TLR4 signaling for IFN-β expression (thus in STAT-1 activation), as compared to C57BL/6 mice, resistant to this parasite. Also importantly, our findings indicate that macrophages may indeed be the major source of IFN-β in *L. major* infection *in vivo*, as previously suggested [53]. The difference between C57BL/6 and BALB/c in the TLR4-IFN-β-STAT-1-iNOS pathway in the absence of adaptive responses is a novel finding that substantiates previous conclusions that early mechanisms involving TLR4, IFN-β and NO [52]–[55], [58] are fundamental to host resistance.

A central aspect of the present findings is the correlation between the resistant/susceptible mouse phenotypes with the intrinsic ability of their macrophages to produce NO, regardless of any ongoing adaptive Th1/Th2 response. *In vivo*, with slight amounts of PAMP/cytokines, C57BL/6, but not BALB/c, macrophages may kill a pathogen via NO, far ahead of T cell influence. The differences observed here may also account for C57BL/6 and BALB/c macrophages skill to distinctly affect subsequent production of Th1 or Th2 cytokines [3]. This correlation implicates macrophages as potential early contributors to the outcome of the infection by decreasing/increasing pathogen burdens while the adaptive immune response is in progress, and by skewing this response in a Th1/Th2 direction, consolidating host resistance/susceptibility.

## Supporting Information

Figure S1 **NO production by BALB/c and C57BL/6 macrophages.**
(TIF)

Figure S2 A**rginase expression and activity in BALB/c and C57BL/6 macrophages.**
(TIF)

Figure S3 **iNOS protein and mRNA expression in BALB/c and C57BL/6 macrophages.**
(TIF)

Figure S4 **STAT-1 and NF-κB expression and activation in BALB/c and C57BL/6 macrophages.**
(TIF)

Figure S5 **IFN-β mRNA expression and effect of IFN-β neutralization on NO production and STAT-1 activation in BALB/c and C57BL/6 macrophages**.
(TIF)

Figure S6 **Effect of exogenous IFN-β on NO production and STAT-1 activation in BALB/c and C57BL/6 macrophages.**
(TIF)

## References

[1] Kumar H, Kawai T, Akira S (2011) Pathogen recognition by the innate immune system. Int Rev Immunol 30: 16–34. 21235323 10.3109/08830185.2010.529976

[2] Mosser DM, Edwards JP (2008) Exploring the full spectrum of macrophage activation. Nat Rev Immunol 8: 958–969. 19029990 10.1038/nri2448 PMC2724991

[3] Mills CD, Kincaid K, Alt J, Heilman MJ, Hill AM (2000) M-1/M-2 macrophages and the Th1/Th2 paradigm. J Immunol 164: 6166–6173.10843666

[4] Oswald IP, Afroun S, Bray D, Petit JF, Lemaire G (1992) Low response of BALB/c macrophages to priming and activating signals. J Leukoc Biol 52: 315–322. 1381743 10.1002/jlb.52.3.315

[5] Stenter S, Thuring H, Rollinghoff M, Bogdan C (1994) Tissue expression of inducible nitric oxide synthase is closely associated with resistance to *Leishmania* major. J Exp Med 180: 783–793. 7520472 10.1084/jem.180.3.783 PMC2191630

[6] Dileepan KN, Page JC, Stechschulte DJ (1995) Direct activation of murine peritoneal macrophages for nitric oxide production and tumor cell killing by interferon-gamma. J Interf Cytok Res 15: 387–394. 10.1089/jir.1995.15.387 7648440

[7] Kaushik RS, Uzonna JE, Gordon JR, Tabel H (1999) Innate resistance to *Trypanosoma congolense* infections: differential production of nitric oxide by macrophages from susceptible BALB/c and resistant C57BL/6 mice. Exp Parasitol 92: 131–143. 10366538 10.1006/expr.1999.4408

[8] Jardim IS, Lima-Santos J, Horta MF (1999) Differential production of nitric oxide by murine macrophages from Leishmania-resistant and -susceptible mice strains. Mem Inst Oswaldo Cruz 94 (suppl.): 197.

[9] Zídek Z, Franková D, Boubelík M (2000) Genetic variation in in-vitro cytokine-induced production of nitric oxide by murine peritoneal macrophages. Pharmacogenetics 10: 493–501. 10975603 10.1097/00008571-200008000-00002

[10] Santos JL, Andrade AA, Dias AAM, Bonjardim CA, Reis LFL, et al (2006) Differential sensitivity of C57BL/6 (M-1) and BALB/c (M-2) macrophages to the Stimuli of IFN-γ/LPS for the production of NO: correlation with iNOS mRNA and protein expression. J Interf Cytok Res 26: 682–688. 10.1089/jir.2006.26.682 16978073

[11] Horta MF, Mendes BP, Roma EH, Noronha FS, Macêdo JP, et al (2012) Reactive oxygen species and nitric oxide in cutaneous leishmaniasis. J Parasitol Res 2012: 203818. 22570765 10.1155/2012/203818 PMC3337613

[12] Liew FY, Millott S, Parkinson C, Palmer RM, Moncada S (1990) Macrophage killing of *Leishmania* parasite *in vivo* is mediated by nitric oxide from L-arginine. J Immunol 144: 4794–4797. 2351828

[13] Green SJ, Nacy CA, Meltzer MS (1991) Cytokine-induced synthesis of nitrogen oxides in macrophages: a protective host response to *Leishmania* and other intracellular pathogens. J Leukoc Biol 50: 93–103. 2056250 10.1002/jlb.50.1.93

[14] Jardim IS, Lima-Santos J, Horta MF, Ramalho-Pinto FJ (1997) Inhibition of the production of nitric oxide impairs cytotoxicity of macrophages to *Leishmania amazonensis*. Mem Inst Oswaldo Cruz 92 (suppl.): 217.

[15] Sacks D, Noben-Trauth N (2002) The immunology of susceptibility and resistance to *Leishmania major* in mice. Nat Rev Immunol 2: 845–858. 12415308 10.1038/nri933

[16] Tacchini-Cottier F, Weinkopff T, Launois P (2012) Does T helper differentiation correlate with resistance or susceptibility to infection with *L. major*? Some insights from the murine model. Front Immunol 3: 32. 22566916 10.3389/fimmu.2012.00032 PMC3342012

[17] Trant GMC, Lacerda TLS, Carvalho NB, Azevedo V, Rosinha GM, et al (2010) The *Brucella abortus* phosphoglycerate kinase mutant is highly attenuated and induces protection superior to that of vaccine strain 19 in immunocompromised and immunocompetent mice. Infect Immun 78: 2283–2291. 20194591 10.1128/IAI.01433-09 PMC2863508

[18] Macedo GC, Magnani DM, Carvalho NB, Bruna-Romero O, Gazzinelli RT, et al (2008) Central role of MyD88-dependent dendritic cell maturation and proinflammatory cytokine production to control *Brucella abortus* infection. J Immunol 180: 1080–1087. 18178848 10.4049/jimmunol.180.2.1080

[19] Hibbs Jr JB, Taintor RR, Vavrin Z, Rachlin EM (1988) Nitric oxide: a cytotoxic activated macrophage effector molecule. Biochem Biophys Res Commun 157: 87–94. 3196352 10.1016/s0006-291x(88)80015-9

[20] Noda T, Amano F (1997) Differences in nitric oxide synthase activity in a macrophage-like cell line, RAW264.7 cells, treated with lipopolysaccharide (LPS) in the presence or absence of interferon-γ (IFN-γ): possible heterogeneity of iNOS activity. J Biochem 121: 38–46. 9058189 10.1093/oxfordjournals.jbchem.a021566

[21] Overbergh L, Valckx D, Waer M, Mathieu C (1999) Quantification of murine cytokine mRNAs using real time quantitative reverse transcriptase PCR. Cytokine javascript:AL_get (this, ‘jour’, %0d%0a'Cytokine.’); 11: 305–312. 10.1006/cyto.1998.0426 10328870

[22] Cardoso CR, Teixeira G, Provinciatto PR, Godoi DF, Ferreira BR, et al (2008) Modulation of mucosal immunity in a murine model of food-induced intestinal inflammation. Clin Exp Allergy 38: 338–349. 18005184 10.1111/j.1365-2222.2007.02866.x

[23] Corraliza IM, Campo ML, Soler G, Modolell M (1994) Determination of arginase activity in macrophages: a micromethod. J Immunol Methods 174: 231–235. 8083527 10.1016/0022-1759(94)90027-2

[24] Gangster RW, Geller DA (2000) Molecular regulation of inducible nitric oxide synthase. In: J Ignarro, editor.Nitric oxide - Biology and Pathobiology. Academic Press, San Diego, CA. pp. 129–156.

[25] Lau JF, Horvath CM (2002) Mechanisms of type I interferon cell signaling and STAT-mediated transcriptional responses. Mt Sinai J Med 69: 156–168. 12035075

[26] Deng W, Ohmori Y, Hamilton TA (1996) LPS does not directly induce STAT activity in mouse macrophages. Cell Immunol 170: 20–24. 8660795 10.1006/cimm.1996.0129

[27] Lu Y-C, Yeh W-C, Ohashi PS (2008) LPS/TLR4 signal transduction pathway. Cytokine 42: 145–151. 18304834 10.1016/j.cyto.2008.01.006

[28] Rossol M, Heine H, Meusch U, Quandt D, Klein C, et al (2011) LPS-induced cytokine production in human monocytes and macrophages. Clin Rev Immunol 31: 379–446. 10.1615/critrevimmunol.v31.i5.20 22142165

[29] Fujihara M, Ito N, Pace JL, Watanabe YS, Russell SW, et al (1994) Role of endogenous interferon-β in lipopolysaccharide-triggered activation of the inducible nitric-oxide synthase gene in a mouse macrophage cell line, J774. J Biol Chem 269: 12773–12778. 7513694

[30] Gao JJ, Filla MB, Fultz MJ, Vogel SN, Russell SW, et al (1998) Autocrine/paracrine IFN-αβ mediates the lipopolysaccharide-induced activation of transcription factor Stat1α in mouse macrophages: pivotal role of Stat1α in induction of the inducible nitric oxide synthase gene. J Immunol 161: 4803–4810. 9794412

[31] Bogdan C (2001) Nitric oxide and the immune response. Nat Immunol 2: 907–916. 11577346 10.1038/ni1001-907

[32] Oswald IP, Wynn TA, Sher A, James SL (1994) NO as an effector molecule of parasite killing: modulation of its synthesis by cytokines. Comp Biochem Physiol Pharmacol Toxicol Endocrinol 108: 11–18. 7520338 10.1016/1367-8280(94)90083-3

[33] Pindado J, Balsinde J, Balboa MA (2007) TLR3-dependent induction of nitric oxide synthase in RAW 264.7 macrophage-like cells via a cytosolic phospholipase A_2_/cycooxygenase-2 pathway. J Immunol 179: 4821–4828. 17878381 10.4049/jimmunol.179.7.4821

[34] Yamamoto M, Sato S, Hemmi H, Hoshino K, Kaisho T, et al (2003) Role of adaptor TRIF in the MyD88-independent toll-like receptor signaling pathway. Science 301: 640–643. 12855817 10.1126/science.1087262

[35] Stuehr DJ, Marletta MA (1985) Mammalian nitrate biosynthesis: mouse macrophages produce nitrite and nitrate in response to *Escherichia coli* lipopolysaccharide. Proc Natl Acad Sci USA 82: 7738–7742. 3906650 10.1073/pnas.82.22.7738 PMC391409

[36] Mizel SB, Honko AN, Moors MA, Smith PS, West AP (2003) Induction of macrophage nitric oxide production by Gram-negative flagellin involves signaling via heteromeric Toll-like receptor 5/Toll-like receptor 4 complexes. J Immunol 170: 6217–6223. 12794153 10.4049/jimmunol.170.12.6217

[37] Buzzo CL, Campopiano JC, Massis LM, Lage SL, Cassado AA, et al (2010) A novel pathway for inducible nitric-oxide synthase activation through inflammasomes. J Biol Chem 285: 32087–32095. 20702413 10.1074/jbc.M110.124297 PMC2952210

[38] Mosmann TR, Cherwinski H, Bond MW, Giedlin MA, Coffman RL (1986) Two types of murine helper T 477 cell clone. Definition according to profiles of lymphokine activities and secreted 478 proteins. J Immunol 136: 2348–2357. 2419430

[39] Heinzel FP, Sadick MD, Holaday BJ, Coffman RL, Locksley RM (http://www.ncbi.nlm.nih.gov/pubmed?term=%22Locksley%20RM%22%5BAuthor%5D1989) Reciprocal expression of interferon gamma or interleukin 4 during the resolution or progression of murine leishmaniasis. Evidence for expansion of distinct helper T cell subsets. J Exp Med 169: 59–72. 2521244 10.1084/jem.169.1.59 PMC2189187

[40] Locksley RM, Scott P (http://www.ncbi.nlm.nih.gov/pubmed?term=%22Scott%20P%22%5BAuthor%5D1991) Helper T-cell subsets in mouse leishmaniasis: induction, expansion and effector function. Immunol Today. 12: A58–A61. 10.1016/S0167-5699(05)80017-9 1829891

[41] Reiner SL, Locksley RM (1993) Cytokines in the differentiation of Th1/Th2 CD4+ subsets in leishmaniasis. J Cell Biochem 53: 323–328. 7905485 10.1002/jcb.240530409

[42] Liew FY, Xu D, Chan WL (1999) Immune effector mechanism in parasitic infections. Immunol Lett 65: 101–104. 10065634 10.1016/s0165-2478(98)00131-x

[43] Laskay T, Diefenbach A, Röllinghoff M, Solbach W (1995) Early parasite containment is decisive for resistance to *Leishmania major* infection. Eur J Immunol 25: 2220–2227. 7664785 10.1002/eji.1830250816

[44] Diefenbach A, Schindleer H, Donhauser N, Lorenz E, Laskay T, et al (1998) Type 1 interferon (IFN-α/β) and type 2 nitric oxide synthase regulate the innate immune response to a protozoan parasite. Immunity 8: 77–87. 9462513 10.1016/s1074-7613(00)80460-4

[45] Zhang X, Alley EW, Russell SW, Morrison DC (1994) Necessity and sufficiency of beta interferon for nitric oxide production in mouse peritoneal macrophages. Infect Immun 62: 33–40. 8262648 10.1128/iai.62.1.33-40.1994 PMC186064

[46] Thomas KE, Galligan CL, Newman RD, Fish EN, Vogel SN (2006) Contribution of interferon-β to the murine macrophage response to the toll-like receptor 4 agonist, lipopolysaccharide. J Biol Chem 281: 31119–31130. 16912041 10.1074/jbc.M604958200

[47] Toshchakov V, Jones BW, Perera P-Y, Thomas K, Cody MJ, et al (2002) TLR4, but not TLR2, mediates IFN-β-induced STAT-1α/β-dependent gene expression in macrophages. Nat Immunol 3: 392–398. 11896392 10.1038/ni774

[48] Cunha FQ, Moncada S, Liew FY (1992) Interleukin-10 (IL-10) inhibits the induction of nitric oxide synthase by interferon-γ in murine macrophages. Biochem Biophys Res Commun 182: 1155–1159. 1371674 10.1016/0006-291x(92)91852-h

[49] Green SJ, Mollouk S, Hoffman SL, Meltzer MS, Nacy CA (1992) Cellular mechanisms of nonspecific immunity to intracellular infection: cytokine-induced synthesis of toxic nitrogen oxides from L-arginine by macrophages and hepatocytes. Immunol Lett 25: 15–19. 10.1016/0165-2478(90)90083-3 2126524

[50] Hohmann HP, Remy R, Pöschl B, van Loon AP (1990) Tumor necrosis factors-alpha and -beta bind to the same two types of tumor necrosis factor receptors and maximally activate the transcription factor NF kappa B at low receptor occupancy and within minutes after receptor binding. J Biol Chem 265: 15183–15188. 2168404

[51] Iyer SS, Amir AG, Cheng G (2010) Lipopolysaccharide-mediated IL-10 transcriptional regulation requires sequentical induction of type I IFNs and IL-27 in macrophages. J Immunol 185: 6599–6607. 21041726 10.4049/jimmunol.1002041 PMC4103176

[52] Bogdan C, Röllinghoff M, Diefenbach A (2000) The role of nitric oxide in innate immunity. Immunol Rev 173: 17–26. 10719664 10.1034/j.1600-065x.2000.917307.x

[53] Mattner J, Wandersee-Steinhäuser A, Pahl A, Rölinghoff M, Majeau GR, et al (2004) Protection against progressive leishmaniasis by IFN-β. J Immunol 172: 7574–7582. 15187137 10.4049/jimmunol.172.12.7574

[54] Kropf P, Freudenberg MA, Modolell M, Prece HP, Herath S, et al (2004) Toll-like receptor 4 contributes to efficient control of infection with the protozoan parasite *Leishmania major* . Infect Immun 72: 1920–1928. 15039311 10.1128/IAI.72.4.1920-1928.2004 PMC375159

[55] Whitaker SM, Colmenares M, Pestana KG, McMachon-Pratt D (2008) *Leishmania pifanoi* proteoglycolipid complex P8 induces macrophage cytokine production through toll-like receptor 4. Infect Immun 76: 2149–2156. 18299340 10.1128/IAI.01528-07 PMC2346673

[56] Assis RR, Ibraim IC, Noronha FS, Turco SJ, Soares RP (2012) Glycoinositolphospholipids from *Leishmania braziliensis* and *L. infantun*: modulation of innate immune system and variations in carbohydrate structure. PLoS Neg Trop Dis 6: e1543. 10.1371/journal.pntd.0001543 PMC3289616 22389743

[57] Ibraim IC, Assis RR, Pessoa NL, Campos MA, Melo MN, et al (2013) Two biochemically distinct lipophosphoglycans from Leishmania braziliensis and Leishmania infantum trigger different innate immune responses in murine macrophages. Parasit Vectors 6: 54. 23497381 10.1186/1756-3305-6-54 PMC3606350

[58] Nagai T, Devergne O, Mueller TF, Perkins DL, van Seventer JM, et al (2003) Timing of IFN-β exposure during human dendritic cell maturation and naïve Th cell stimulation has contrasting effects on Th1 subset generation: a role for IFN-β-mediated regulation of IL-12 family cytokines and IL-18 in naïve Th cell differentiation. J Immunol 171: 5233–5243. 14607924 10.4049/jimmunol.171.10.5233

