# Supplementary figures and images for: A Defective TLR4 Signaling for IFN-β Expression Is Responsible for the Innately Lower Ability of BALB/c Macrophages to Produce NO in Response to LPS as Compared to C57BL/6
Source: PLoS One. 2014 Jun 9;9(6):e98913. doi: 10.1371/journal.pone.0098913 (PMC4049611; doi:10.1371/journal.pone.0098913)

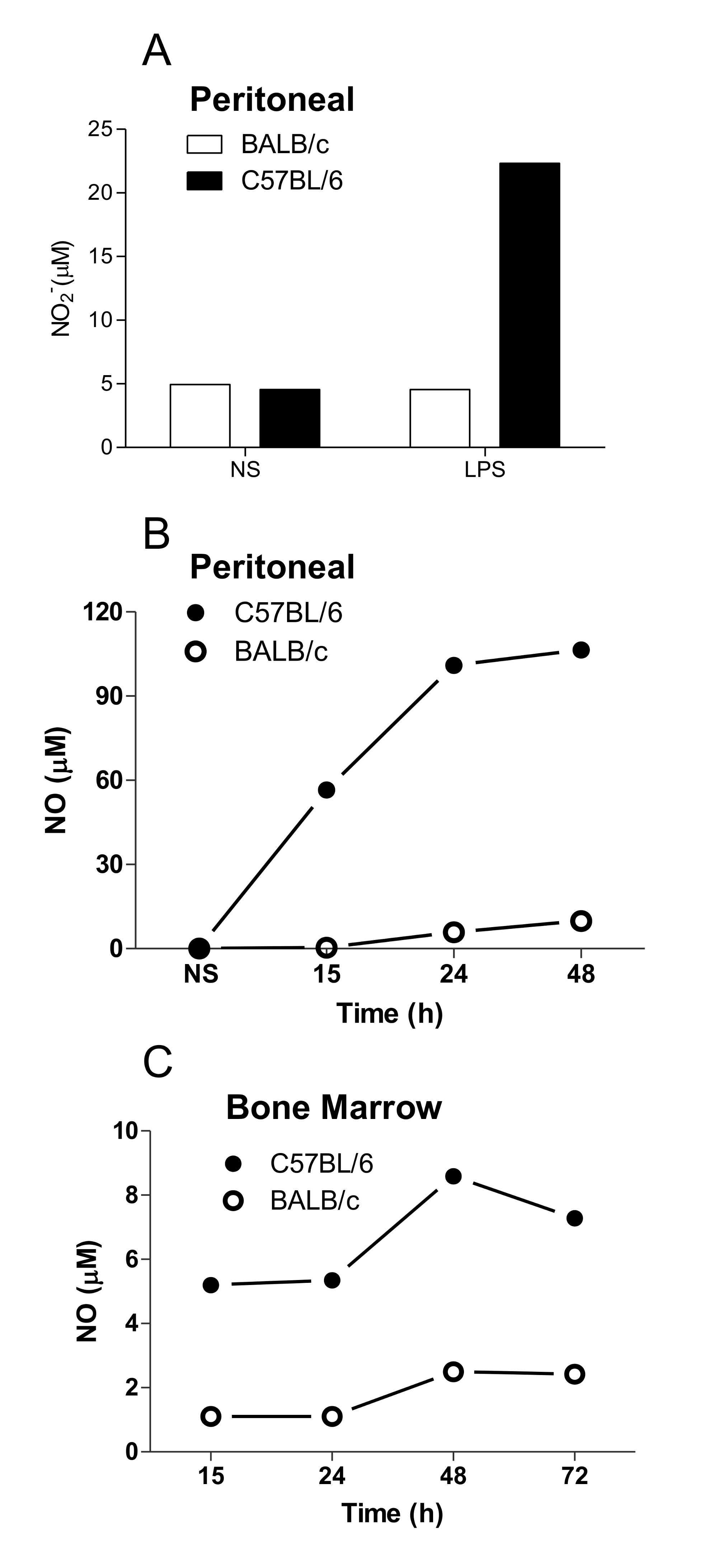

Supplement: Figure S1 — NO production by BALB/c and C57BL/6 macrophages. (TIF) [file pone.0098913.s001.tif]

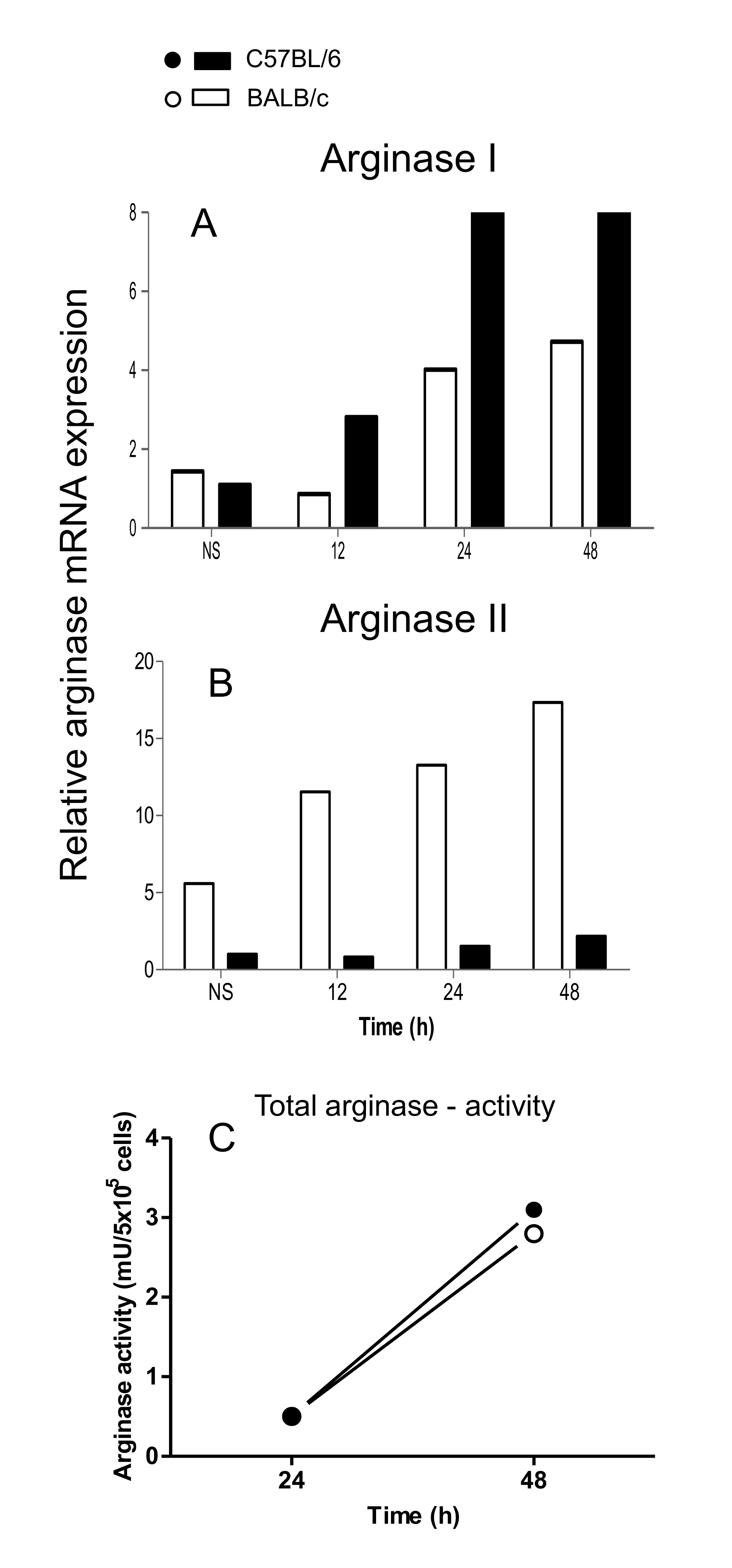

Supplement: Figure S2 — Arginase expression and activity in BALB/c and C57BL/6 macrophages. (TIF) [file pone.0098913.s002.tif]

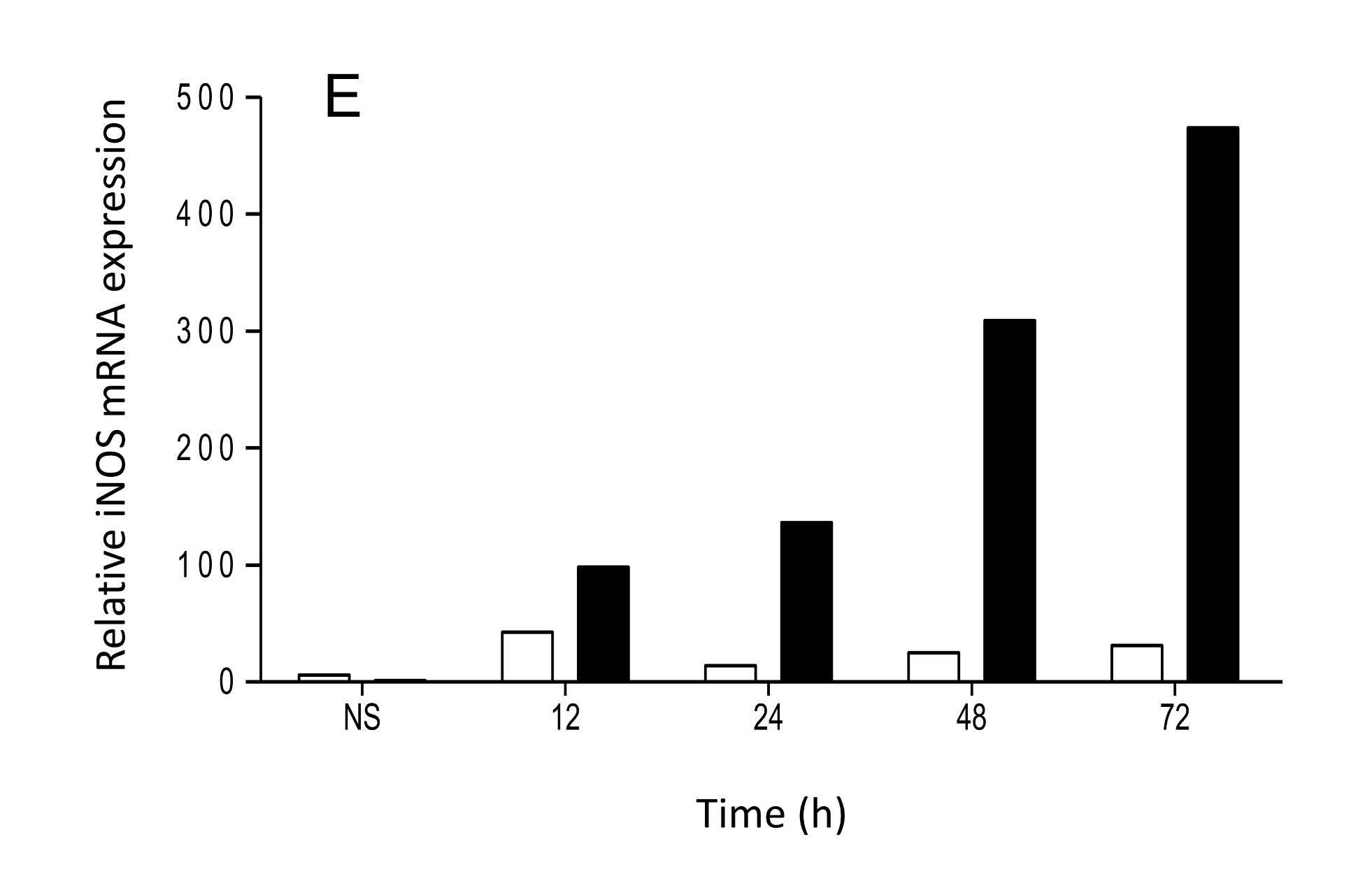

Supplement: Figure S3 — iNOS protein and mRNA expression in BALB/c and C57BL/6 macrophages. (TIF) [file pone.0098913.s003.tif]

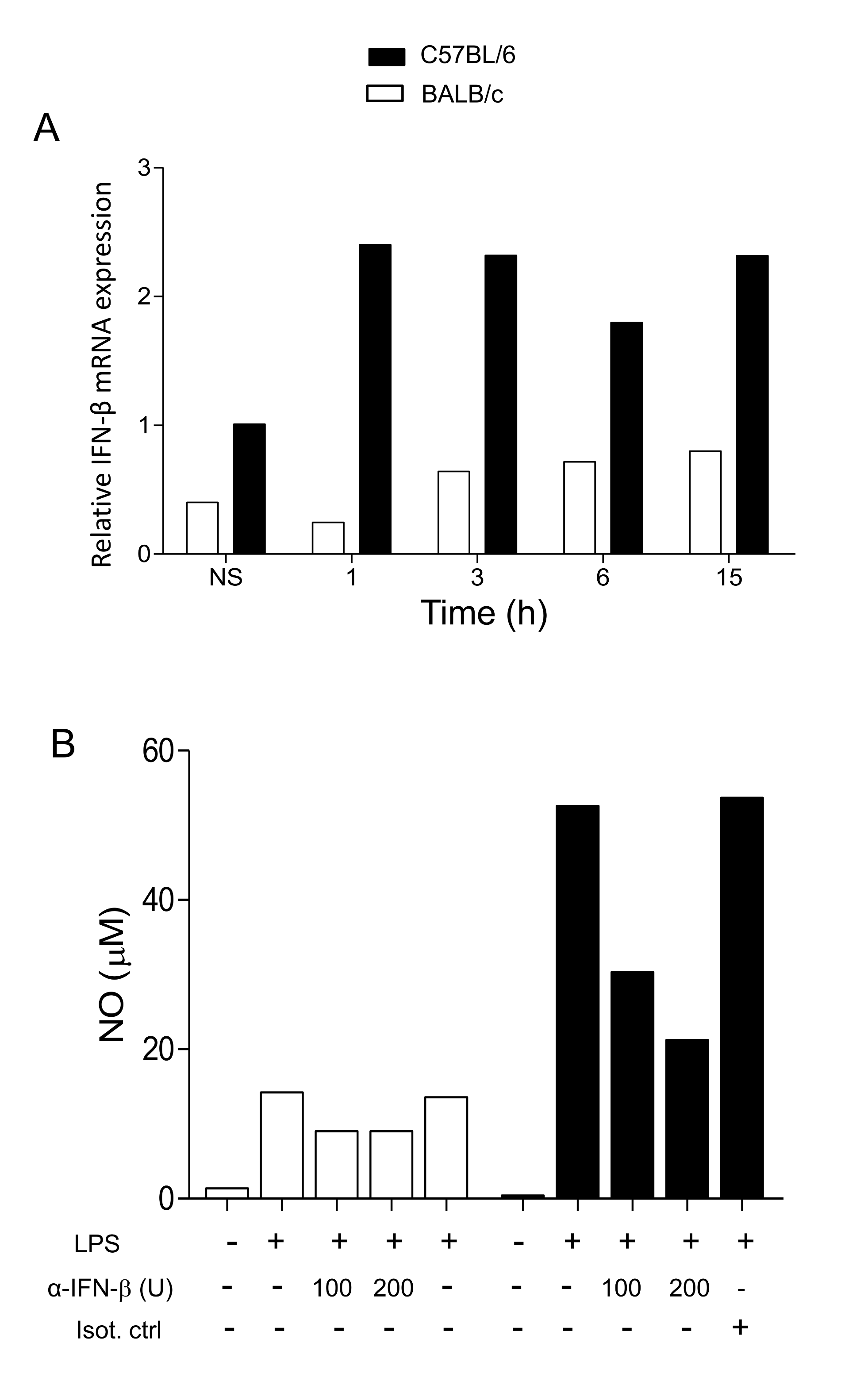

Supplement: Figure S4 — STAT-1 and NF-κB expression and activation in BALB/c and C57BL/6 macrophages. (TIF) [file pone.0098913.s004.tif]

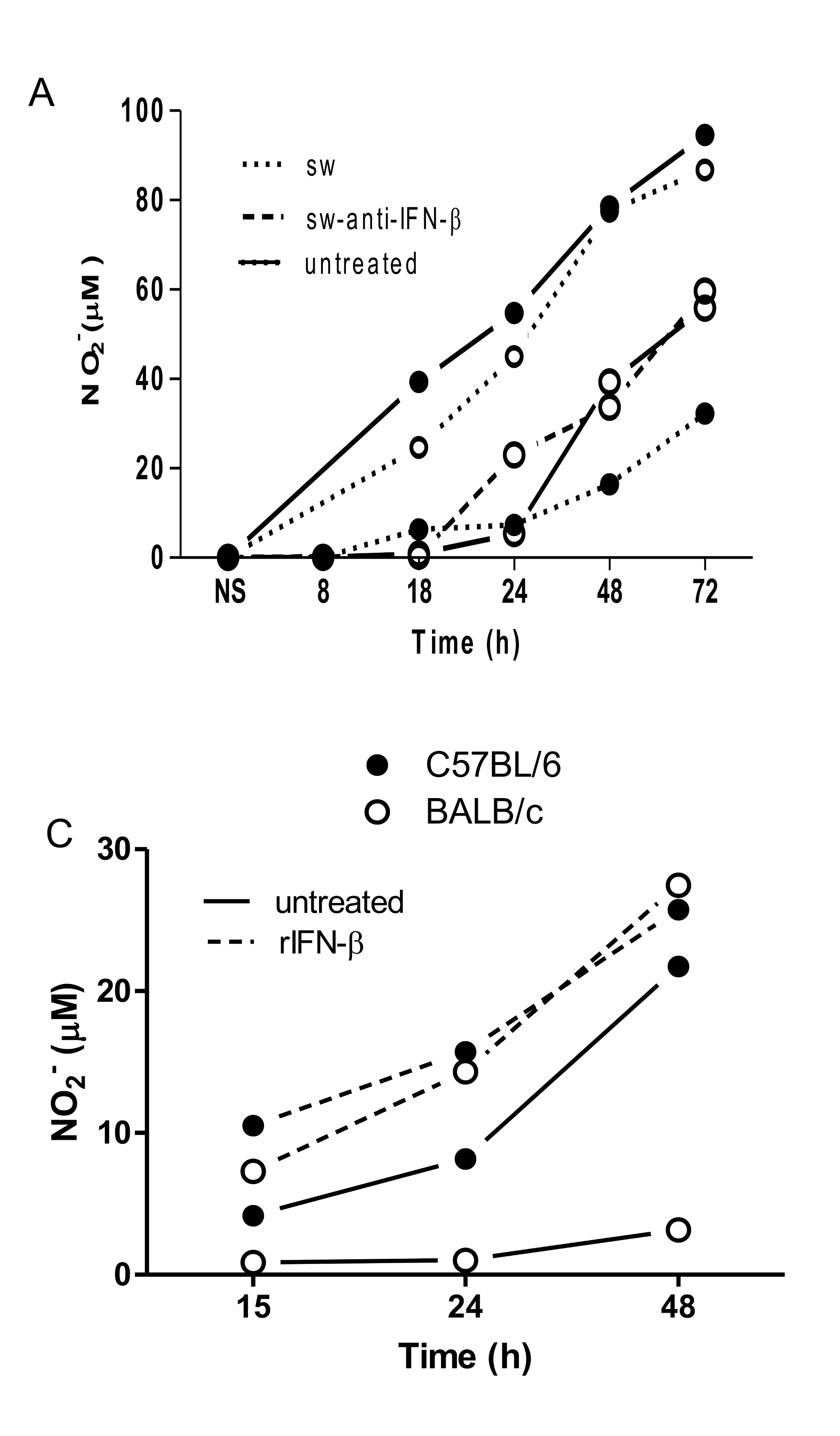

Supplement: Figure S5 — IFN-β mRNA expression and effect of IFN-β neutralization on NO production and STAT-1 activation in BALB/c and C57BL/6 macrophages. (TIF) [file pone.0098913.s005.tif]

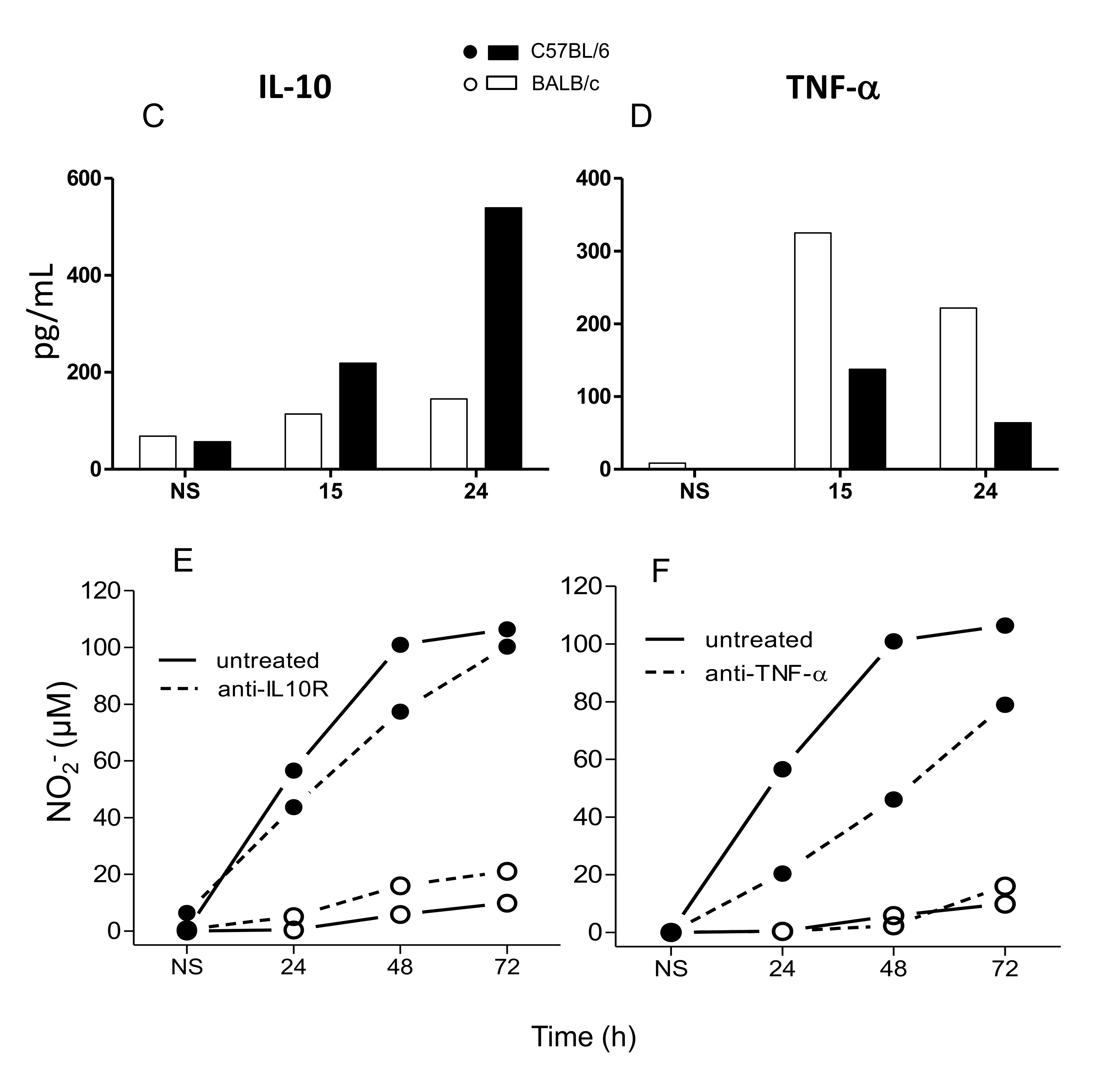

Supplement: Figure S6 — Effect of exogenous IFN-β on NO production and STAT-1 activation in BALB/c and C57BL/6 macrophages. (TIF) [file pone.0098913.s006.tif]
